# Crystallization of nanopore-confined imidazolium ionic liquids probed by temperature-resolved *in situ* grazing-incidence wide angle X-ray scattering (GIWAXS)

**DOI:** 10.1039/d5na00509d

**Published:** 2025-07-28

**Authors:** Yuxin He, M. Arif Khan, Andrew D. Drake, Joshua Garay, Aniruddha Shirodkar, Stephen Goodlett, Joseph Strzalka, Folami Ladipo, Barbara L. Knutson, Stephen E. Rankin

**Affiliations:** a University of Kentucky, Department of Chemical and Materials Engineering 177 F.P. Anderson Tower Lexington Kentucky 40506-0046 USA bknut2@uky.edu stephen.rankin@uky.edu; b University of Kentucky, Department of Chemistry 125 Chemistry/Physics Building Lexington Kentucky 40506-0055 USA; c X-ray Science Division, Argonne National Laboratory Argonne Illinois 60439 USA

## Abstract

The crystallization behavior of ionic liquids (ILs) 1-butyl-3-methylimidazolium [BMIM] hexafluorophosphate [PF_6_] and chloride [Cl] is investigated upon confinement in 2.3 or 8.2 nm diameter silica nanopore arrays, along with the effects of covalently modifying the pore walls with 1-(3-trimethoxysilylpropyl)3-methylimidazolium [TMS-MIM]^+^ groups. *In situ* grazing-incidence wide angle X-ray scattering (GIWAXS) is performed during heating from as low as −110 °C to room temperature. Partially ordered “nanodomains” are observed in both ILs in the bulk molten state, but they are disrupted by nanoconfinement. Melting point depression consistent with capillary effects is observed for [BMIM][PF_6_] in 2.3 nm pores. However, the melting point is elevated for [BMIM][PF_6_] in 8.2 nm pores, which provide sufficient space to stabilize the crystalline phase. For [BMIM][Cl], crystallization is observed only in 8.2 nm bare silica pores, but the melting point is severely depressed. Tethering with IL-like [TMS-MIM^+^] also promotes the crystallization of [BMIM][PF_6_], resulting in elevated melting points. The combined effects of a larger pore size and pore surface tethering on [BMIM][PF_6_] result in a single stable crystal phase that persists from −140 °C to 25 °C (*vs.* the bulk melting point of −11 °C). These results show that when ILs are used in confined systems, complex crystallization behavior can emerge depending on the counterion, pore size, and surface modification that require consideration of ion layering in the confined space in addition to surface free energy effects.

## Abstract 2

Nanoconfinement effects on [BMIM][PF_6_] crystallization in 8.2 nm pores studied *via* GIWAXS show transition temperatures shifts due to a trade-off between surface energy and ion layering at pore surfaces.

## Graphical abstract

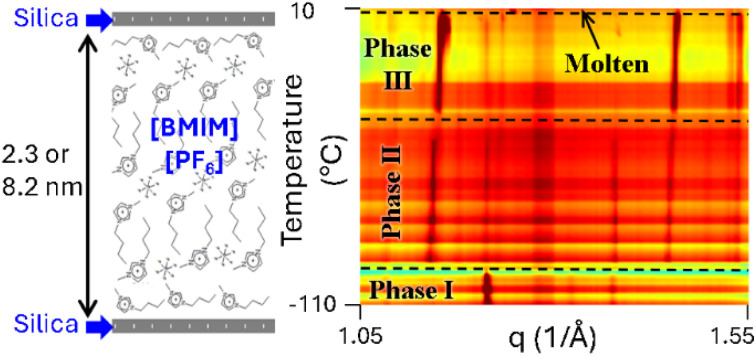

## Introduction

Ionic liquids (ILs) are salts with melting points near ambient conditions – usually below 100 °C. They are of interest for a wide range of applications because of their low volatility, good thermal stability, high ionic conductivity and versatile properties. ILs are also known as tunable solvents for a variety of inorganic, organic and polymeric materials^1,2^ and as media for catalytic reactions.^3–5^ Although catalysis and separations using bulk ILs are intensively studied and well established,^6–9^ many applications benefit from using nanopore-confined (“nanoconfined”) ILs, either to reduce the cost and quantity of IL required, to prevent contamination of contacting phases with ILs, or to ease separation. A further benefit of nanoconfinement is using interfacial effects to further tune structure, solvation, transport, and catalytic properties.

Supported IL (SIL) catalysts are among the most-studied nanoconfined systems, in which ILs are chemically or physically immobilized in solid porous matrices, such as polymers, silicas, and carbon materials.^10–14^ Studies of successful hydroformylation, hydrogenation, and other reactions using supported ILs have been reported.^6,15–17^ Confined ionic liquids also play important roles in emerging applications such as electrochemical energy storage,^18–20^ CO_2_ fixation,^21–24^ and supported IL membranes.^25–29^ Numerous experimental and computational studies show evidence that nanoconfinement of ILs causes significant changes in thermal, electrochemical, transport, and molecular assembly behavior.^12,30–35^

Crystal phase transition behavior is among the properties of ILs that are affected by confinement.^30,36,37^ Crystallization is an important aspect of IL physical chemistry because as complex, self-assembling fluids, they are prone to exhibit unusual solidification and melting behavior.^38–40^ Moreover, crystallization of ILs directly affects ionic conductivity, molecular transport and catalyst effectiveness, which may dictate operating temperatures for SILs.^41–44^ Early observations of the crystal structures of imidazolium salts, a commonly used family of ILs, revealed polymorphism in the crystal structures of 1-butyl-3-methylimidazolium chloride ([BMIM][Cl]).^38,45–47^ Subsequently, polymorphism was reported for 1-butyl-3-methylimidazolium hexafluorophosphate ([BMIM][PF_6_]).^41,48^ Because the crystallographic behavior of these two ILs has been extensively investigated in bulk, [BMIM][Cl] and [BMIM][PF_6_] will be the two ILs of choice for this study.

Two competing effects are anticipated for nanoconfined ILs. On one hand, Gibbs–Thomson theory predicts that a molecular liquid should experience melting temperature (*T*_m_) depression under confinement due to the interfacial energy at the pore surface and that the degree of *T*_m_ depression is inversely related to the pore diameter.^49^ This effect has been reported for ILs confined in nanoporous silica “ionogels” prepared by gelation in the presence of the IL.^50^ In small pores (<10 nm), evidence for crystallization has been reported to be lost, suggesting that strong interactions with the pore wall may prevent the formation of an ordered solid.^50–52^ However, researchers have observed the opposite for ILs confined in other materials. The second competing effect of confinement of ILs is caused by layering of the liquids near interfaces. Chen *et al.* confined [BMIM][PF_6_] in carbon nanotubes and obtained a composite with extreme *T*_m_ elevation by about 200 °C compared to unconfined [BMIM][PF_6_] due to stabilization of the crystalline phase by layering at the pore wall.^30^ Another study of the same IL confined in mesoporous silica particles also showed significant *T*_m_ elevation after treatment with compressed gas.^53^ Atomic force microscopy (AFM) studies have shown the formation of solid-like layers near rough silica surfaces for [BMIM] bis(trifluoromethanesulfonyl)imide.^54^ Within the same nanoporous silica support, *T*_m_ elevation or depression has been shown to depend on the type of IL being confined.^55^ All of these observations show that nanoconfinement effects on crystallization are complex, and more study of the effects of variables such as pore size and surface chemistry is needed.

Mesoporous silica materials are selected as support matrices in the study because of their tunable porosity and stable chemical and thermal properties.^56,57^ The films are synthesized using a surfactant-templated sol–gel method, specifically evaporation-induced self-assembly by dip coating onto silicon wafer substrates.^58–60^ This is one of many approaches available to synthesise silica with vertically oriented mesopores^61–63^ and creates ideal structures for investigating confinement effects by grazing incidence X-ray scattering techniques. Nanoporous thin films with two different pore diameters, 2.3 nm and 8.2 nm, are prepared using cetyltrimethylammonium bromide (CTAB)^64^ and Pluronic P123 triblock copolymer^65^ as structure directing agents, respectively. These supports also provide the opportunity to observe the effects of surface modification with tethered ionic liquid-like functional groups. These grafting groups can be used to stabilize ILs in the pores,^66,67^ similar to what has been studied for CO_2_/CO separation and catalysis.^11,13,68^ Romanos and coworkers previously showed evidence that grafted imidazolium ionic liquids crystallize in mesoporous silica pores, whereas physically entrapped ILs do not.^11,68^ This inspired us to further investigate the impacts of tethering on nanoconfined ILs, specifically using the organosilane IL 1-(3-trimethoxysilylpropyl)-3-methylimidazolium chloride ([TMS-MIM][Cl]) to graft onto surface silanol groups.

Here, the effects of nanoconfinement in silica and [TMS-MIM][Cl]-modified silica are investigated using *in situ* grazing incidence wide angle X-ray scattering (GIWAXS). When investigating crystallization of imidazolium ILs, it is common to use differential scanning calorimetry (DSC) to determine overall phase transition temperatures and a combination of X-ray diffraction (XRD), Raman or other techniques for structural information.^30,37,69–71^ However, the crystallization of imidazolium ILs is complicated due to the large ion sizes, asymmetry, and the possibility of multiple carbon chain conformations.^72^ Because ILs are complex fluids, cold crystallization and polymorphism are common. Temperatures for the onset of crystallization and transitions between polymorphs have been found to be history dependent, as are the final solid-to-liquid transition temperatures. [BMIM][PF_6_], for example, has been reported to exhibit melting points that vary in the range of 7.9 ± 3.8 °C when prepared and measured under different conditions in previous studies^36,41,48,70,73–75^ (Table S1 in the SI), so our own studies of unconfined ILs were conducted to set a baseline for the current series of experiments. The high intensity synchrotron source used here provides the temporal resolution needed for a comprehensive picture regarding both transition temperatures and crystal structures by temperature-resolved *in situ* GIWAXS. It also helps to distinguish single and co-existing crystal phases as a function of temperature. This study takes advantage of our ability to create silica films with accessible, vertically oriented mesopore channels of varying sizes to provide novel insights into how nanoconfinement affects the crystallization behavior of [BMIM][Cl] and [BMIM][PF_6_] by *in situ* X-ray scattering.

## Materials and methods

Methods for synthesis of silica thin films with 8.2 nm pores by templating with Pluronic surfactant P123, silica films with 2.3 nm pores by templating with cetyltrimethylammonium bromide, and modification of their surfaces by attachment of 1-(3-trimethoxysilylpropyl)3-methylimidazolium chloride [TMS-MIM][Cl] follow the same procedures that were previously reported by He *et al.*^76,77^ The materials and methods for their synthesis are detailed in the SI. The materials and methods for the preparation and characterization of unconfined and nanopore-confined ILs are presented here.

### Materials

Acetonitrile (anhydrous, 99.8%), toluene (anhydrous, 99.8%), 1-butyl-3-methylimidazolium hexafluorophosphate ([BMIM][PF_6_], ≥98%), and 1-butyl-3-methylimidazolium chloride ([BMIM][Cl], ≥98%) for ionic liquid loading into nanopores were obtained from Sigma-Aldrich.

### X-ray photoelectron spectroscopy (XPS) depth profile of the [TMS-MIM][Cl] tethered silica film

A K-Alpha XPS instrument (Thermo Scientific) was employed to measure the compositions of the elements C, N, Si, and O using ion gun etching with an electron flood gun for charge compensation. A survey scan was first performed and then Si and O were analyzed with 10 high resolution scans while C and N were analyzed with 15 scans presuming small amounts relative to the other two elements. The Ar^+^ ion gun power was set at 500 eV for 12 increments of etching in total between the readings. The etching time was 10 seconds for the first 4 intervals and 200 seconds for the remaining 8 intervals. *Avantage* software (Thermo Scientific) was used for both data acquisition and analysis. The atomic percentages of the elements were calculated based on the electron density *versus* binding energy peak areas using *Avantage* and plotted with respect to the etching time.

### [BMIM][Cl] recrystallization and drying

[BMIM][Cl] was purified by recrystallization in a nitrogen purged glove bag to eliminate ambient humidity.^78^ About 5 g of [BMIM][Cl] was mixed with just enough acetonitrile to dissolve the IL after 30 min of stirring. Then, about 10 drops of toluene were added to the solution. The final solution was cooled in a freezer at −18 °C overnight. After that, white crystals of [BMIM][Cl] formed. They were washed with cold toluene after filtration. Finally, the crystals were heated at 100 °C in a vacuum oven overnight to eliminate toluene residue and moisture taken up during brief exposure to ambient air. The vacuum was released by the introduction of high-purity nitrogen gas right before deposition of the IL.

### [BMIM][PF_6_] drying

Approximately 2 ml of the purchased IL was transferred to a glass vial and vacuum dried at 100 °C overnight to remove residual moisture. Then the vacuum was released by introducing high-purity nitrogen gas and kept in a nitrogen purged environment until deposition.

### IL deposition

Both ILs were deposited on five different substrates for comparison: bare flat silicon (Si) wafers (unconfined IL), P123-templated silica films on Si wafers (confined IL), P123-templated silica films tethered with [TMS-MIM][Cl] (Tconfined IL) and CTAB-templated silica films with and without the same tethering on glass slides. For unconfined ILs, an 8 mm diameter and 0.5 mm depth silicone isolator (Grace Bio-Labs) was placed on a Si wafer and 50 μL of IL was placed in the isolator to form a “pool” of IL. For confined ILs, a small drop of each IL was placed on the silica films and then covered with a piece of 125 μm thick Kapton film. The Kapton film was gently scraped with a ruler wrapped with a Kimwipe® to spread and squeeze out excess IL from the edge of the Kapton film. This was to minimize the IL sitting between the Kapton film and silica film. Since [BMIM][Cl] is moisture sensitive, this step was carried out in a nitrogen-filled glove bag. [BMIM][PF_6_] is not as hygroscopic as [BMIM][Cl], so it was quickly deposited under ambient air.

### *In situ* GIWAXS measurement with varying temperature

GIWAXS measurements were conducted using Beamline 8-ID-E at the Advanced Photon Source at the Argonne National Laboratory. The synchrotron X-ray wavelength was 0.114 nm with a beam size of 800 μm × 4 μm. The *in situ* cold crystallization was carried out in a vacuum chamber at a pressure of 0.01 torr and the temperature was controlled using a Linkam HFSX350 stage (Linkam Scientific). After evacuation, the samples went through a cooling cycle from 25 °C to −140 °C (−120 °C for unconfined [BMIM][PF_6_] and −130 °C for confined [BMIM][PF_6_]) with a temperature ramp of 10 °C min^−1^. This was followed by a 10 min hold, heating back to 25 °C at 10 °C min^−1^, a 10 min hold, and cooling a second time to the minimum temperature at 10 °C min^−1^. After the second cooling cycle and holding for 10 min, the samples were slowly heated at 0.3 °C min^−1^ for GIWAXS measurements. Two seconds of beam exposures were used for measurements about every five minutes.

### Sample temperature *vs.* Linkam HFSX350 stage temperature

The temperature at the surface of silica films deposited on both Si wafers and glass slides was measured to determine the sample temperature with an external resistance temperature detector (RTD Pt-111, Lake Shore Cryotronics) and compared to the temperature read from the Linkam stage. This sample temperature on a Si wafer was measured with the RTD epoxied to the top surface of a mesoporous silica film (Scheme S1) while lowering the temperature of the Linkam stage from room temperature to −150 °C at 10 °C min^−1^ and then raising it to 280 °C at 10 °C min^−1^. The temperature curves do not differ significantly over the temperature range of −140 °C to 200 °C (Fig. S1). For silica films deposited on glass slides, the temperature was lowered from room temperature to −140 °C, maintained for 10 minutes and then raised back to room temperature at the same ramp rate as above. The lowest sample surface temperature only went down to about −105 °C when the Linkam temperature reading was −140 °C and there was about a three-minute temperature response delay below −65 °C when cooling but a much smaller offset during heating at 10 °C min^−1^ (Fig. S2). The temperature ramp rate during GIWAXS measurement is much lower (0.3 °C min^−1^ heating compared to 10 °C min^−1^), so it is reasonable to also assume that the Linkam stage temperature reading is true to the sample surface temperature when it was above −105 °C and that −105 °C represents the minimum known temperature even though the Linkam stage reached a lower temperature for glass slides. A caveat regarding this comparison is that thermal contraction may have affected the measurement. During the measurement, the RTD sensor on the sample surface slightly lifted the glass slide, causing loss of contact of the glass slide with the Linkam temperature stage. Thus, the lowest temperature of −105 °C is the worst-case scenario and is used to represent a conservative upper bound on the range of temperatures investigated for glass substrates.

### Crystallographic analysis

The 2D GIWAXS patterns were integrated azimuthally and reduced to intensity with respect to scattering vector *q* (Å^−1^) using the MATBLAB based software GIXSGUI.^79^ The values of *q* in reciprocal space were then converted to 2*θ* (°) values with a wavelength of 1.54 Å (Cu Kα as the X-ray source) for easier comparison with the literature. A background spectrum of the Kapton film was subtracted from the confined and Tconfined samples. The crystal structures of the samples were determined using TOPAS software (Bruker) by fitting lattice parameters starting from the IL crystal structures of Dibrov *et al.*^48^ for [BMIM][PF_6_] (COD# 2014366) and Holbrey *et al.*^45^ for [BMIM][Cl] (COD# 7103776). Atom occupancy and coordinates as well as temperature factors were fixed during fitting using a Pearson VII peak shape function.

## Results and discussion

### Mesoporous thin film synthesis and characterization

The mesopore structures of the two types of silica films (with 2.3 nm and 8.2 nm pores) were verified by grazing-incidence small angle scattering (GISAXS) to be vertically oriented, hexagonal close packed nanopore channels. The orthogonal pore orientation in P123-templated films was achieved by confining the silica film between two surfaces modified with crosslinked P123, giving a film about 180 nm thick after curing and calcination.^58–60^ The vertically aligned channels in CTAB-templated films were obtained by doping the silica matrix with 2 wt% titania to soften the matrix and allow merging of pores in the vertical direction due to thermal contraction of the films during calcination, as recently described by our group.^76^ These films were 90 nm thick as determined by profilometry. The characterization results closely matched those recently reported for a study of redox probe transport in similar films on fluorine-doped tin oxide coated glass.^77^ TEM images of pieces of both films delaminated from their substrates are presented in Fig. S3, along with pore size distributions measured from these images using *ImageJ* software. The pore sizes and standard deviations are 8.2 ± 0.5 nm for P123-templated films and 2.3 ± 0.5 nm for CTAB-templated films.

To examine the accessibility of the pore pathway and confirm successful [TMS-MIM][Cl] tethering in the pores of the films, Tconfined films were analyzed by XPS depth profiling. The composition profiles of films with both 2.3 nm and 8.2 nm pores are presented in the SI (Tables S2 and S3, respectively) as a function of cumulative ion etching time. Because the length of the etching steps varied, the absolute depth is not known, but it is clear from the atomic percentages of C and N that a uniform layer of [TMS-MIM][Cl] was tethered onto the pore wall throughout the entire thickness of both types of silica films. A constant composition of both C and N is found from the top surface until the point that the silicon signal dominates (when the Si substrate is reached) or the glass substrate (SiO_2_) becomes the primary composition.

### Crystallization and melting of [BMIM][PF_6_]

**Fig. 1:**
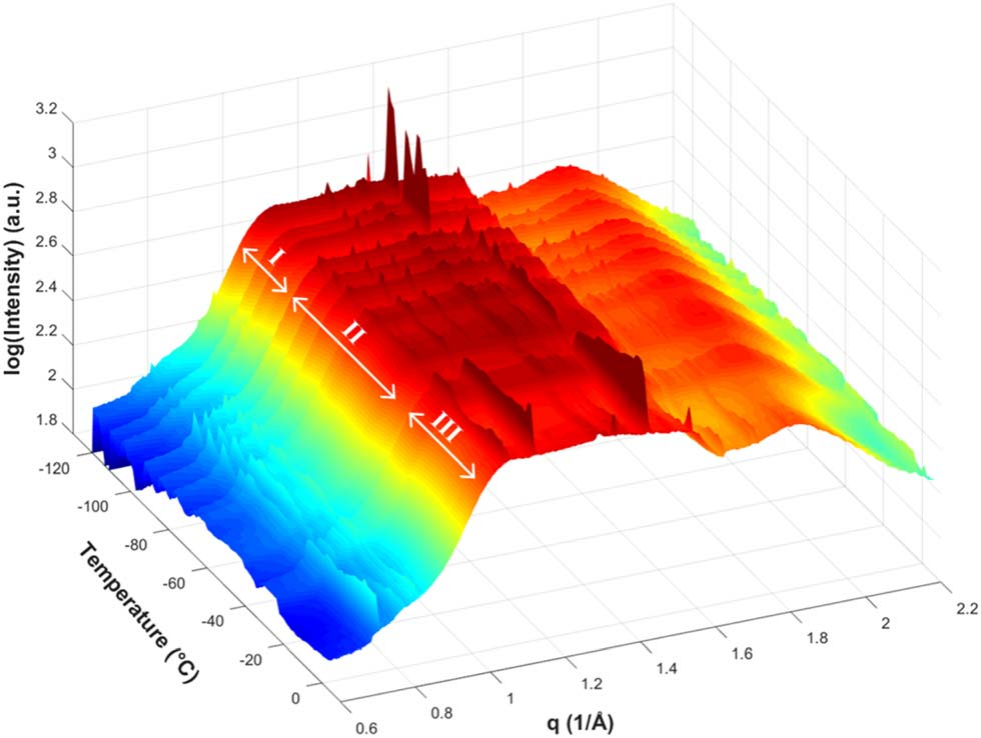
3D plot of integrated GIWAXS data as a function of temperature prior to background subtraction for [BMIM][PF_6_] confined in 8.2 nm nanopores.

Having established that the films have the expected vertical nanopore structure and that pore tethering was successful, we now discuss the results of temperature-resolved GIWAXS for confined and unconfined ILs. Representative 2D GIWAXS patterns and their analysis are presented in the Supplemental Information but only integrated 1D patterns are shown in the main text. Fig. 1 shows an example of the full series of 1D GIWAXS patterns for confined [BMIM][PF_6_] from *in situ* measurement during slow heating from −120 °C. The ridges in the direction of increasing temperature are caused by sample alignment issues, which were resolved in the patterns presented in the text by background subtraction. Three regions of sharp peaks from crystalline ionic liquid can be identified, labeled as I, II and III. These correspond to three polymorphs of confined [BMIM][PF_6_] (discussed further below). At the highest temperatures in Fig. 1, the sharp peaks from crystalline IL disappear, indicating melting of the IL. Background subtraction was performed for data presentation and analysis from this point forward to eliminate the interference from the Kapton film and reflection from the substrate. A sample GIWAXS background is presented in the SI (Fig. S4).

#### Unconfined [BMIM][PF_6_]

**Fig. 2:**
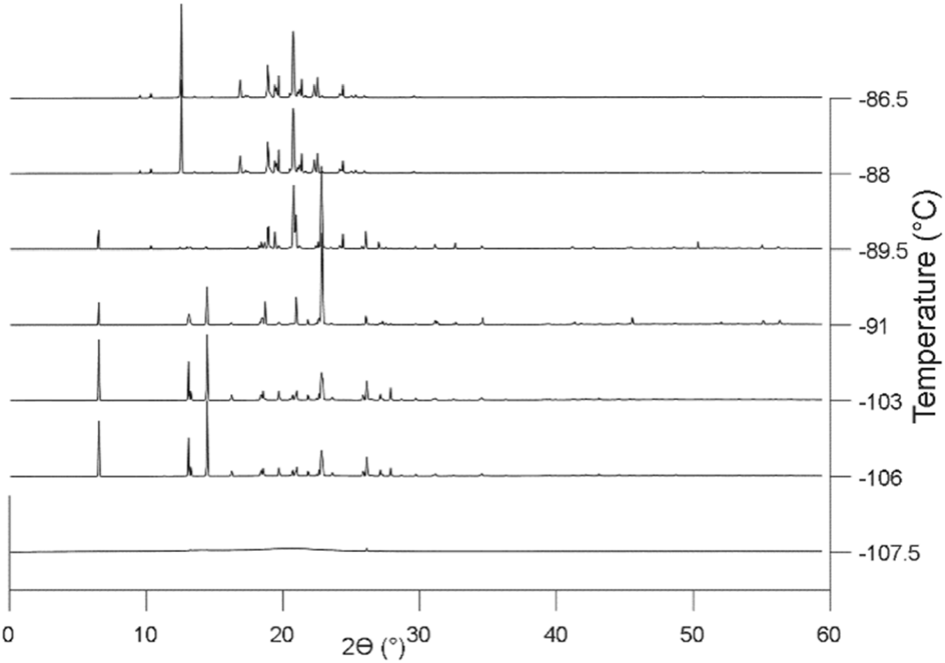
Representative 1D GISAXS patterns of unconfined [BMIM][PF_6_] showing crystallization from the amorphous subcooled state to phase I at −106 °C and then transformation to phase II starting at −91 °C. The GIWAXS pattern at 25 °C is subtracted as the background. For this and other sets of 1D patterns, the vertical axis of each pattern represents integrated intensity (a.u.). Temperatures are labelled for all 1D patterns.

**Fig. 3:**
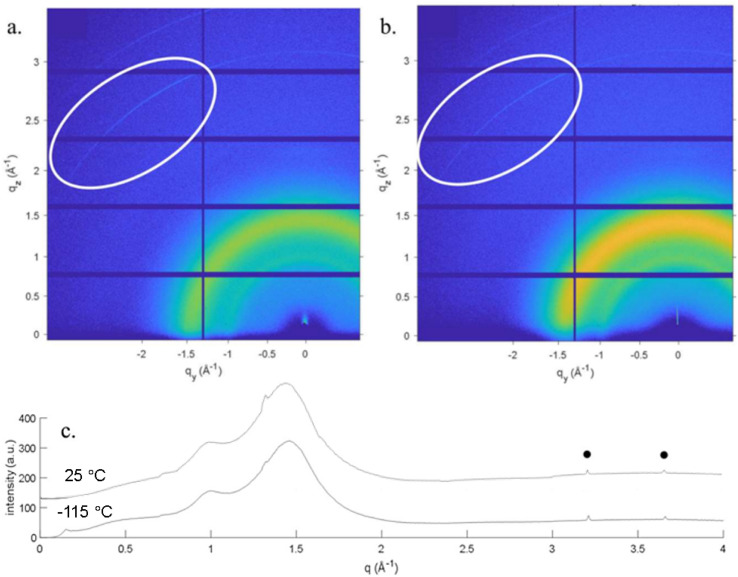
2D GIWAXS pattern of unconfined [BMIM][PF_6_] at (a) −115 °C and (b) 25 °C and (c) corresponding 1D integrated patterns indicating the existence of nanodomains (marked with filled circles in the 1D patterns) before and after forming solid crystal phases.

Before diving into the complexities of confined systems, GIWAXS results for bulk [BMIM][PF_6_] supported only by a bare Si wafer are discussed. This unconfined [BMIM][PF_6_] did not crystallize during cooling to −120 °C, but crystallization began when the temperature was raised to −106 °C (Fig. 2). This cold crystallization behavior has been noted previously in DSC studies of [BMIM][PF_6_],^41,80^ and the lack of a crystal pattern at −107.5 °C in Fig. 2 is representative of this behavior. However, before crystallization is observed, the GIWAXS pattern shows indications of a short-range local structure, or “nanodomains”, with small *d*-spacings (Fig. 3a and c). The pair of reflections corresponding to the nanodomains are low in intensity, most likely because of their small size and concentration. These nanodomains are also present in the liquid state; above the melting temperature, the same short-range nanodomains were observed as in supercooled [BMIM][PF_6_] and maintained at the end of measurement at 25 °C (Fig. 3b and c). This agrees with numerous molecular dynamic simulation studies showing heterogeneous ordered domains in ILs,^72,81,82^ as well as XRD results of Triolo *et al.* for other 1-alkyl-3-methyl-imidazolium-based salts.^83^ However, these GIWAXS results provide direct experimental evidence of nanodomains in amorphous and liquid [BMIM][PF_6_].

Unconfined [BMIM][PF_6_] is found to form two identifiable stable polymorphs and the phase transition temperature between the two is −91 °C (Fig. 2). The second polymorph has a similar diffraction pattern to that reported by Triolo^70^ and Choudhury^41^ at around −90 °C, but with minor differences in unit cell parameters. The melting point is −11 °C, which is lower than reported values ranging from 1.9 °C to 11 °C (Table S1).^36,41,48,73,80^ Different thermal histories (including heating and cooling rates) can result in the observation of different polymorphs and phase transition temperatures, which is common among studies of ILs^70,80^ For example, Triolo *et al.* observed two polymorphs noted as cry.I and cry.II. Cry.I occurred when [BMIM][PF_6_] was kept at −13 °C for a few hours after quenching and then cooling cry.I to −27 °C caused it to transform into cry.II. However, this behavior changed with a different heating procedure. Cry.II appeared first at −53 °C when heating from −113 °C and transformed into cry.I when the heating continued to −21 °C.^70^

The current study was not designed to produce [BMIM][PF_6_] single crystals, so the small crystallite size can contribute to melting point depression according to the Gibbs–Thomson effect (discussed below).^49,84^ For example, the crystallite size of [BMIM][PF_6_] was calculated to be only 5.3 nm at −88 °C using peak widths and the Scherrer equation.^85^ Another common cause for melting point depression is the presence of impurities. In the cases of ILs, water is usually the most common and inevitable impurity. According to Huddleston *et al.*, *T*_m_ was found to be 4 °C and 10 °C for [BMIM][PF_6_] containing 11 700 ppm and 590 ppm of water,^73^ indicating that trace levels of water in [BMIM][PF_6_] are probably not responsible for reducing the melting temperature by more than 20 °C. Because [BMIM][PF_6_] is not highly hygroscopic and was vacuum dried at elevated temperature prior to analysis, it can be concluded that this melting temperature depression was not likely to be caused by water impurities.

#### [BMIM][PF_6_] confined in bare silica nanopores

In confined systems, a sandwiched sample geometry was used for GIWAXS measurements consisting of a Si substrate, a silica nanoporous thin film loaded with IL, and a high X-ray transmittance Kapton film covering the sample. Therefore, it was necessary to isolate contributions to the scattering patterns from the silica nanoporous thin film layer. To do so, the GIWAXS pattern of a confined sample without IL crystallinity was taken and the low-*q* region was expanded. The beam stop is prominent in the resulting image, but the pattern is consistent with the perpendicular HCP structure from the nanoporous silica thin film that was observed by GISAXS (Fig. S4 shows this for a Si wafer and Kapton film, and Fig. S5 with a 2.3 nm, CTAB-templated film). Since we can see the pattern from the ordered silica support at low angles, the current setup is confirmed to give a GIWAXS signal from the confined IL in the silica nanoporous film layer. For the films with 8.2 nm pores, the GIWAXS signal from the nanoporous film was too close to the beam stop to be observed. The X-ray penetration depth of the tethered film was estimated to be about 10.9 nm based on the incidence angle of 0.16° and the atomic percentages from the XPS depth profile of the films (Table S2).^86,87^

**Fig. 4:**
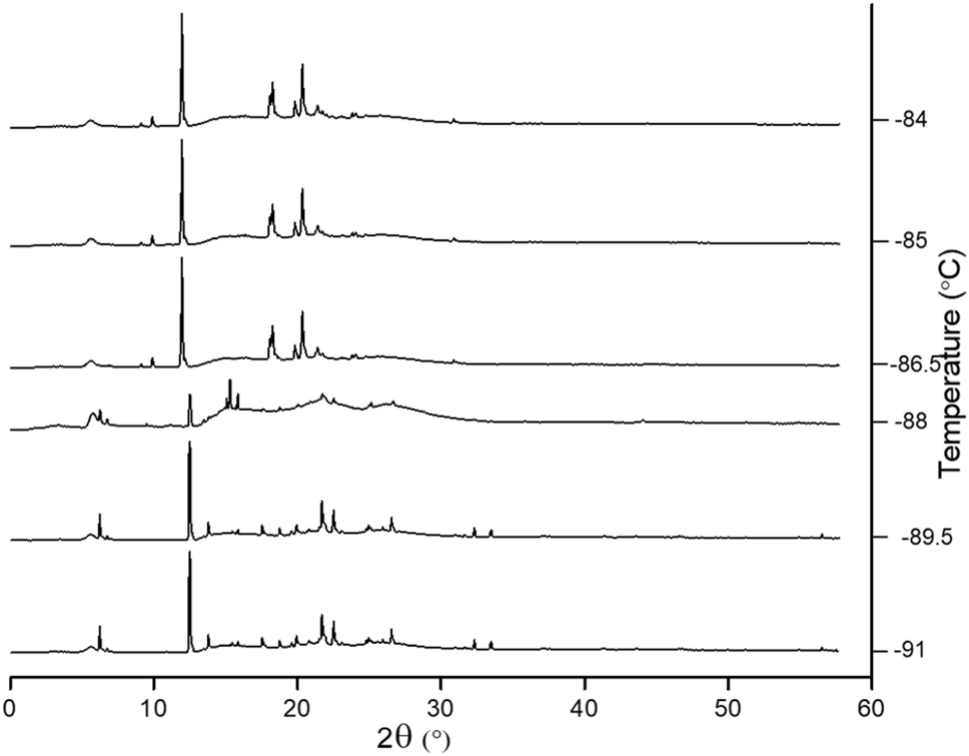
Representative GIWAXS patterns showing the primary crystal phase transition of confined [BMIM][PF_6_] in 2.3 nm silica pores from crystal phase I to phase II starting from −88 °C. Below this temperature, phase I is observed, and phase II is observed above −84 °C until *T*_m_ reaches −20 °C. The vertical axis represents integrated intensity (a.u.).

**Fig. 5:**
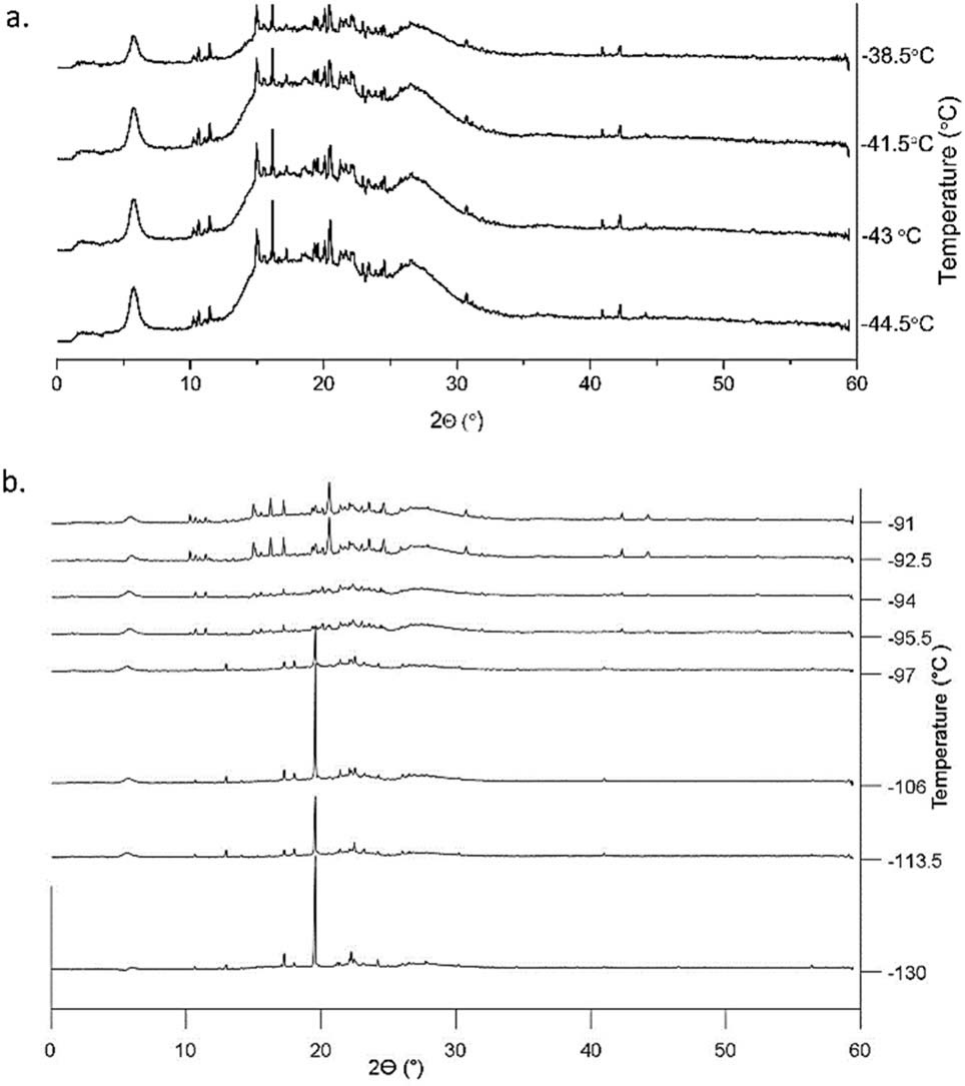
GIWAXS patterns showing the crystal phase transition of confined [BMIM][PF_6_] in 8.2 nm pores (a) from crystal phase II to phase III with the disappearance of the peak around 2*θ* = 17° starting from −41.5 °C; and (b) from crystal phase I to phase II starting from −97 °C. The vertical axis represents integrated intensity (a.u.).

With the confirmation that the X-ray scattering is probing the structure of the nanoporous layer, we begin to address the effects of confinement of [BMIM][PF_6_] in bare silica supports with 2.3 nm and 8.2 nm pores. Confined [BMIM][PF_6_] in both types of films exhibits three polymorphs in the temperature range measured that all have different peak positions and relative intensities compared to the unconfined IL (Fig. 4 and 5). As anticipated, [BMIM][PF_6_] displays complicated crystallization behavior and it changes with pore size. In 2.3 nm pores, the IL crystallizes at the start of the heating ramp into polymorph I and undergoes a transition to polymorph II at −88 °C, close to the transition temperature of the unconfined system. However, in the 2.3 nm pores, [BMIM][PF_6_] melts at −20 °C, which is 9 °C lower than the unconfined IL. This *T*_m_ depression is described by the Gibbs–Thomson equation for confined molecular liquids (eqn (1)).1
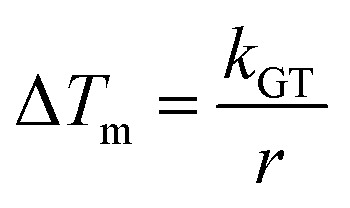
where Δ*T*_m_ is the melting temperature depression (*T*_bulk_ − *T*_confined_) and *r* is the pore radius.^49^*k*_GT_ is a constant depending on liquid properties, pore geometry and pore wall wetting, and is usually positive in sign. The second type of silica film templated with P123 has the same type of pore geometry and pore chemistry, so it should have the same wetting characteristics as [BMIM][PF_6_]. Eqn (1) can then be used to predict that the *T*_m_ of [BMIM][PF_6_] confined in 8.2 nm pores is expected to be higher than when confined in 2.3 nm pores but still lower than that of unconfined [BMIM][PF_6_].

When confined in 8.2 nm silica pores, [BMIM][PF_6_] also crystallizes at the start of the heating ramp into a first polymorph (at −130 °C). Fig. 5b shows that the first phase transition to the second polymorph happened at −97 °C, a lower temperature than in 2.3 nm pores. This second polymorph then transformed into a third crystal phase at around −40 °C (Fig. 5a). While confinement in 8.2 nm pores causes a lower first crystal phase transition temperature than in the bulk IL, the last polymorph of [BMIM][PF_6_] melts at 6.5 °C, experiencing a *T*_m_ elevation by 17.5 °C compared to unconfined [BMIM][PF_6_].

**Fig. 6:**
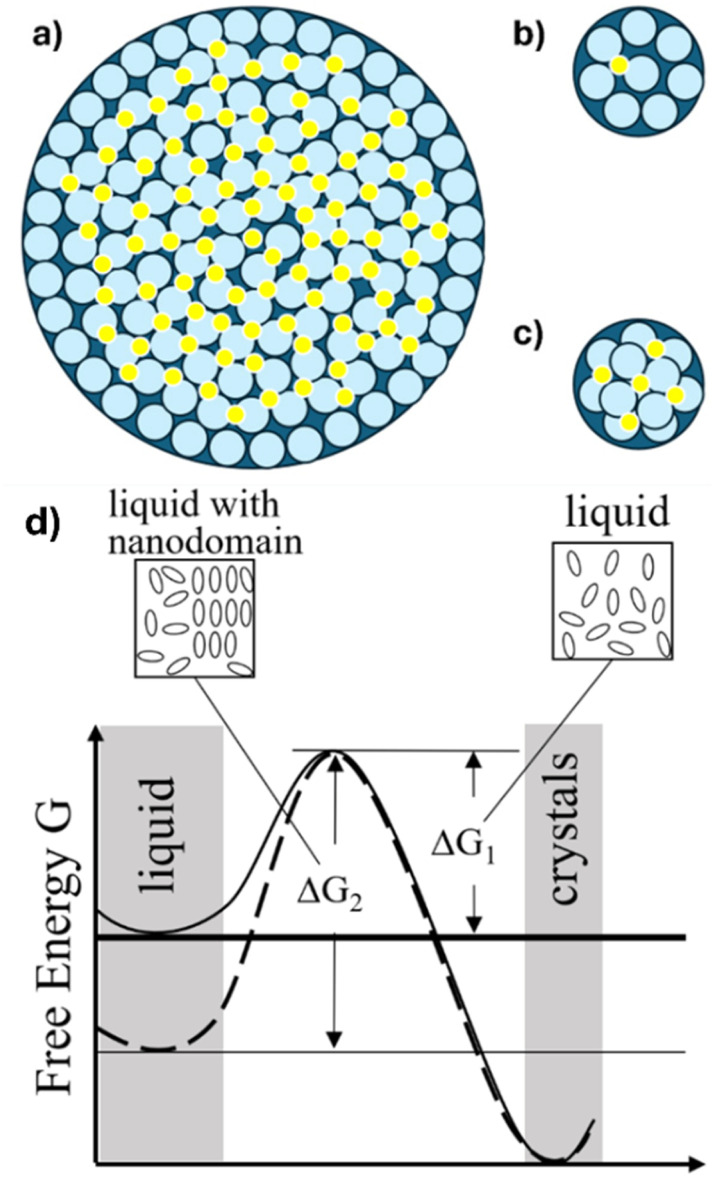
Schematic of packing of [BMIM]^+^ (light blue) and anions (yellow) into (a) an 8.2 nm pore, (b) a single layer in a 2.3 nm pore, and (c) a pair of layers in a 2.3 nm pore. In (a)–(c), counterions are not depicted for [BMIM]^+^ in contact with the pore wall. Panel (d) compares the free energy landscape for crystallization of a randomly oriented liquid with the free energy barrier of Δ*G*_1_ (solid curve) and a liquid with a short range nanodomain such as [BMIM][PF_6_] with a relatively higher free energy barrier of Δ*G*_2_ (dashed curve).

The Gibbs–Thomson equation apparently does not explain the effect of pore size on melting of [BMIM][PF_6_], which calls for an alternative explanation for this behavior. Previously, *T*_m_ elevations were observed when [BMIM][PF_6_] was confined in carbon nanotubes and 2D nanographene sheets.^30,88^ The confined [BMIM][PF_6_] showed significant *T*_m_ elevation in both cases, which was attributed to the promotion of molecular order by nanoconfinement. The Gibbs–Thomson model assumes that the molecular structures of the solid and liquid phases are unaffected by confinement, so it does not capture the possibility that confinement enhances ordering in the liquid phase and therefore stabilizes the solid against melting. In the case of SiO_2_ nanopores, the simulation study of Sha *et al.* suggests an average distance of 0.67 nm between BMIM^+^ ions in layers, consistent with their ionic radius.^40,89^Fig. 6a illustrates an ensemble of molecules of this size (light blue circles) packed into an 8.2 nm pore, allowing 6 concentric rings of molecules to fit. 30% of the [BMIM]^+^ ions are associated with the pore surface and partially displace counterions, so anions (yellow) are shown only for [BMIM]^+^ not associated with the pore wall. In a 2.3 nm pore, Fig. 6b shows that there is room for only one layer of surface-associated [BMIM]^+^ and one additional molecule (87.5% of cations are surface-associated). If a second layer is considered with no pore association in an A–B stacking configuration (Fig. 6c), the surface-associated [BMIM]^+^ reduces to 58.3%.

Fig. 6a–c show that the 8.2 nm pores give more space for [BMIM][PF_6_] to organize, thus showing a stronger nanoconfinement-induced ordering effect (leading to *T*_m_ elevation). This effect may be enhanced by the scaled pore diameter (*D*_pore_/*D*_BMIM^+^_ = 12.2) being close to an integer, thus promoting layering in the liquid state and reducing the thermodynamic driving force for melting. Alternating between molten and frozen states has been reported as pore size varies among a few molecular diameters for an ionic fluid in both charged and neutral pores.^90^ The 2.3 nm pores have *D*_pore_/*D*_BMIM^+^_ = 3.43, which is farther from an integer number of layers and therefore may reduce the impact of pore-induced layering. Fig. 6b suggests that free volume caused by a mismatch between cation radius and pore size may destabilize crystalline phases to promote melting. This can be reconciled with the Gibbs–Thomson equation by noting that for ionic liquids, surface forces oscillate as distances approach a few cation diameters.^91–93^ These correspond to oscillations in interfacial free energy that is proportional to *k*_GT_ in eqn (1), and it can shift positively or negatively with *D*_pore_.

As noted above, unlike unconfined [BMIM][PF_6_], nanoconfined [BMIM][PF_6_] in both 2.3 nm and 8.2 nm pores already crystallizes by the time the system reaches the minimum temperature (≤−105 °C for 2.3 nm pore films on glass slides or −140 °C for 8.2 nm pore films on Si wafers). This may be because the nanodomains found in bulk [BMIM][PF_6_] are disrupted by silica nanoconfinement. Consistent with this hypothesis, no GISAXS features indicating ordered domains are observed in confined [BMIM][PF_6_] after melting. This leaves molten [BMIM][PF_6_] with disordered molecule distances and orientations, which is at a higher free energy state than [BMIM][PF_6_] with ordered nanodomains, as shown in the schematic in Fig. 6d. Therefore, the free energy barrier to crystallization is smaller for confined [BMIM][PF_6_]. This explains the absence of a supercooled liquid state for confined [BMIM][PF_6_]. It is also possible that one or more layers of ions near the pore wall promote heterogeneous nucleation of crystallites and contribute to the depletion of nanodomains from the rest of the fluid.

#### Effects of IL tethering on nanoconfined [BMIM][PF_6_] crystallization

In addition to investigating pore size effects on IL crystallization, the effects of functionalizing the surface with IL-like groups were studied. IL grafting has been used for the purpose of preventing IL leaching, and we have observed that attachment of [TMS-MIM][Cl] to the silica pore surface dramatically changes the permeability of redox probes through nanoporous silica films.^77^ By changing the interactions of the free IL with the surface, tethering is also expected to have significant impacts on the crystallization behavior of ILs because they are in contact with imidazolium groups rather than surface silanol groups.^13^ When [BMIM][PF_6_] was confined in a [TMS-MIM][Cl] tethered silica film with 2.3 nm pores, it underwent similar “cold crystallization” as observed in unconfined [BMIM][PF_6_], where it did not show crystallinity until it was heated slowly to −99 °C (Fig. S6). This did not happen to the IL confined in bare silica films of either pore size, indicating that the tethering with imidazolium groups provides a more bulk-IL-like environment for the confined [BMIM][PF_6_]. As the temperature kept increasing, a second polymorph appeared at −57 °C and coexisted with the first one until there was only the second polymorph remaining at −43.5 °C (Fig. S7). It completely melted at 1 °C (Fig. S8), which is a 12 °C increase compared to the *T*_m_ of unconfined [BMIM][PF_6_] and a 21 °C increase compared to the *T*_m_ of [BMIM][PF_6_] confined in the same type of film but without tethering. This suggests that the [TMS-MIM][Cl] tethering promotes the ordering of [BMIM][PF_6_] in 2.3 nm silica pores.

**Fig. 7:**
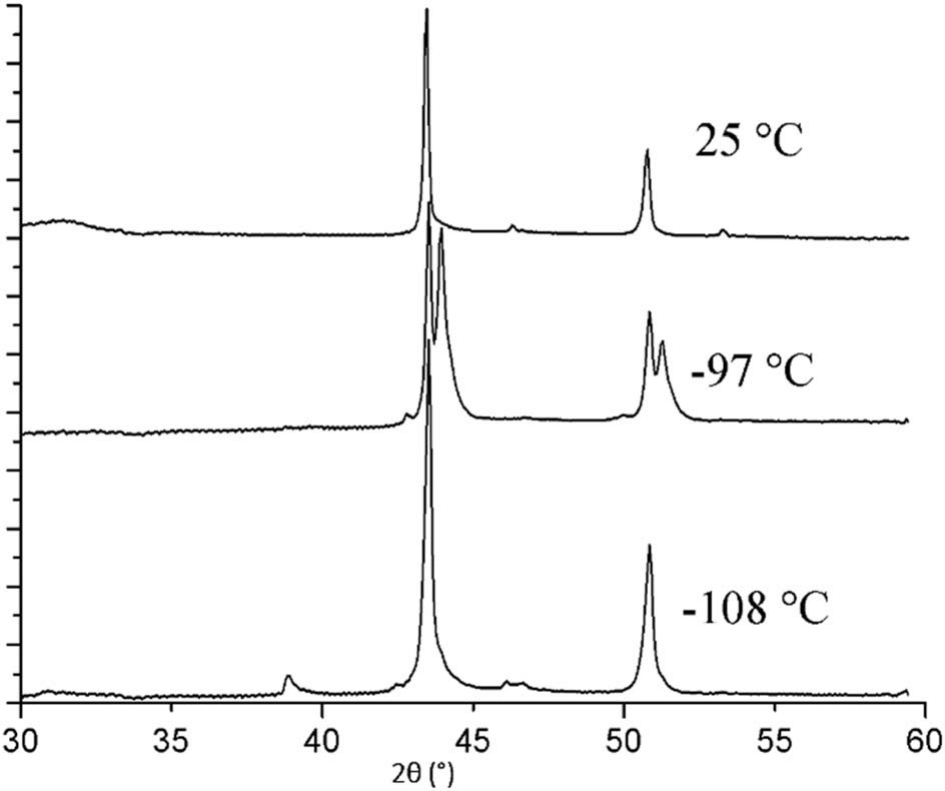
Three representative GISAXS patterns of Tconfined [BMIM][PF_6_] in the mesoporous silica film with 8.2 nm channels throughout the temperature range showing one metastable crystal phase. The vertical axis represents integrated intensity (a.u.).

The promotion of ordering was observed to be even further amplified in the tethered 8.2 nm pores. When [BMIM][PF_6_] was confined in the 8.2 nm nanoporous silica film with tethering, it did not show significant crystallization after two cycles of cooling at 10 °C min^−1^, followed by slow heating at 0.3 °C min^−1^, but exhibited a stable crystal phase after aging for 4 days under nitrogen at room temperature. The crystal phase experienced some *d*-spacing change during the heating process but showed a single stable crystal phase (Fig. 7). The crystal phase did not melt or show indication of reduced diffraction intensity at room temperature and the crystal structure is highly symmetric (as indicated by the small number of peaks in the patterns). It is not uncommon that it takes a few hours to a few days to obtain stable crystal phases in the case of ILs.^38,47,83,94^ Stabilization of the liquid by layering of ions at the pore surface may have played a role in the slow crystal nucleation in the tethered, confined case as well.

Taken together, the *in situ* GIWAXS results presented so far show that [BMIM][PF_6_] exhibits complex crystallization behavior due to cold crystallization, nanosize effects, and confinement-induced ordering. While there is some degree of melting point elevation in bare 8.2 nm silica pores, tethering them with [TMS-MIM][Cl] stabilizes the confined [BMIM][PF_6_] to only one crystal phase due to the combination of molecular ordering promotion effects from both nanoconfinement and surface modification. This can be highly beneficial for the design of stable supported ionic salt systems such as in electrochemical devices, but should be viewed as a warning for systems requiring liquid phase behavior such as catalysis and drug delivery systems. In other words, a room temperature “ionic liquid” may in fact become a crystalline solid under application conditions due to nanopore confinement.

### Crystallization and melting of [BMIM][Cl]

#### Unconfined [BMIM][Cl]

**Fig. 8:**
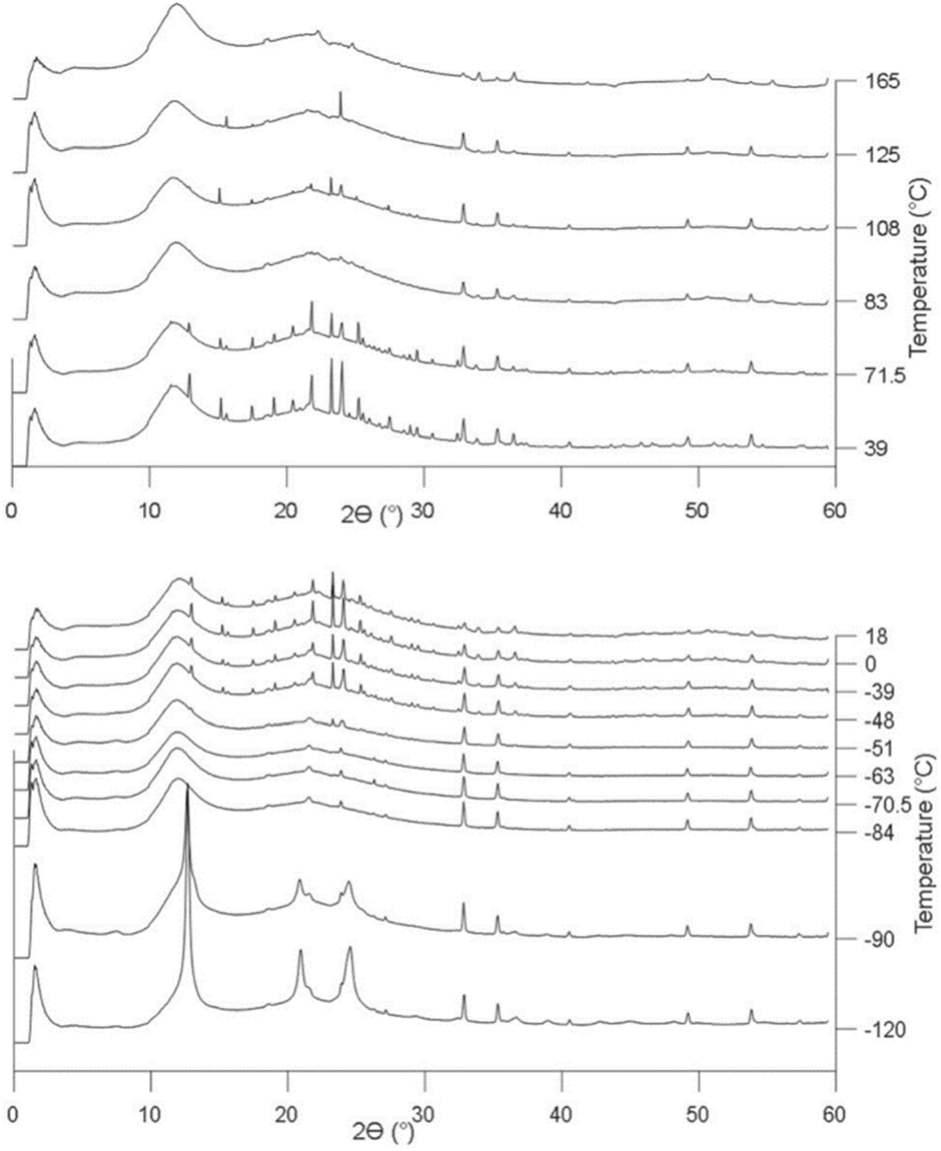
Representative GISAXS pattern of unconfined [BMIM][Cl]. Crystal phase transitions are observed from phase I to the amorphous state at around −84 °C and then from the amorphous state to phase II at around −48 °C (bottom). A melting transition from phase II to the amorphous state then occurs at around 71.5 °C (top). The pattern almost remains the same between −48 °C and 71.5 °C. The vertical axis represents integrated intensity (a.u.).

**Fig. 9:**
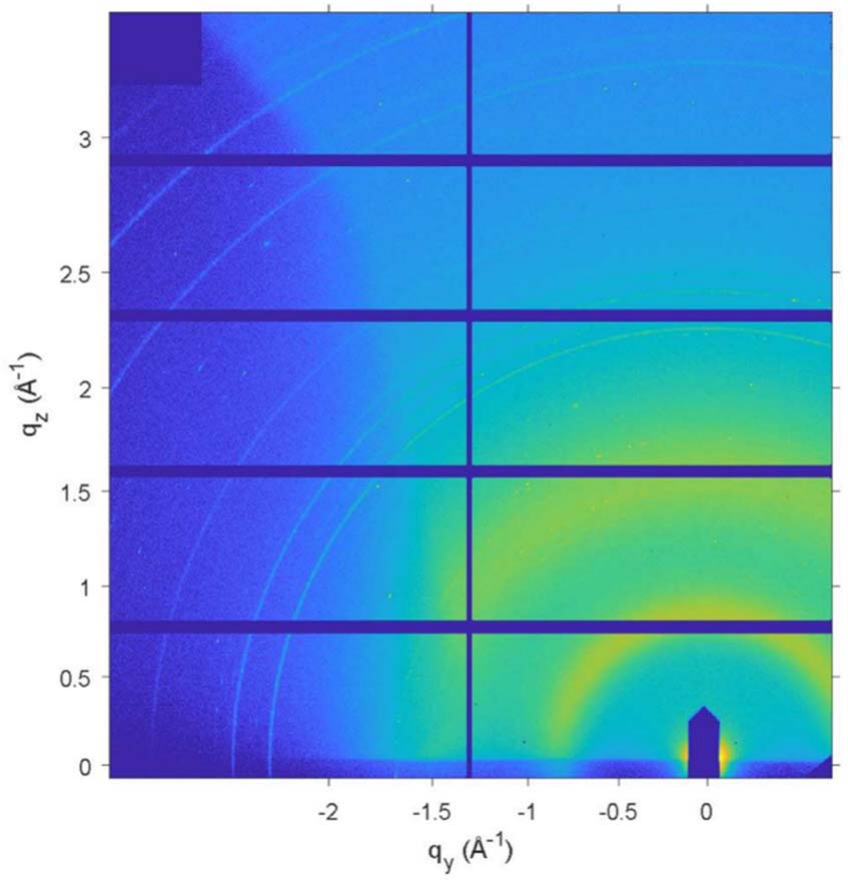
2D GIWAXS pattern of unconfined [BMIM][Cl] at 165 °C indicating the existence of nanodomains well above the melting temperature.

When considering nanoconfinement effects, it is important to recognize that IL properties can be significantly influenced by the counterion.^44,73,95–97^ Thus, a hygroscopic IL with the same cation but an anion smaller in size, [BMIM][Cl], was also investigated to expand our understanding of nanoconfinement. It is believed that the interaction between the cation and anion in [BMIM][Cl] is stronger than that in [BMIM][PF_6_] and this results in [BMIM][Cl] being in a solid form at room temperature.^38,95^ GIWAXS of unconfined [BMIM][Cl] (Fig. 8) gave a *T*_m_ close to reported values at around 70 °C.^47,48,98,99^ In an XRD study of the [BMIM][Cl] structure, Hayashi *et al.* reported two polymorphs.^47^ One of the polymorphs is a metastable form that could be obtained by holding [BMIM][Cl] at −18 °C for two days and the other is stable at −78.5 °C. The unconfined [BMIM][Cl] in this study also exhibits two polymorphs. The first one appeared when the measurement started at −120 °C without having to go through a cold crystallization step and melted at around −84 °C (Fig. 8). The second polymorph emerged at −48 °C and completely melted at around 70 °C. Short range nanodomains were also observed in molten [BMIM][Cl] and during the temperature gap between the two polymorphs. The high-angle reflections associated with the nanodomains were sustained until the end of measurement at 165 °C (Fig. 9). These two polymorphs act similarly to the report of Hayashi *et al.*^47^ in that the two forms appear in different temperature ranges and do not have overlapping peaks.

#### Effects of confinement and tethering on [BMIM][Cl] crystallization and melting

**Fig. 10:**
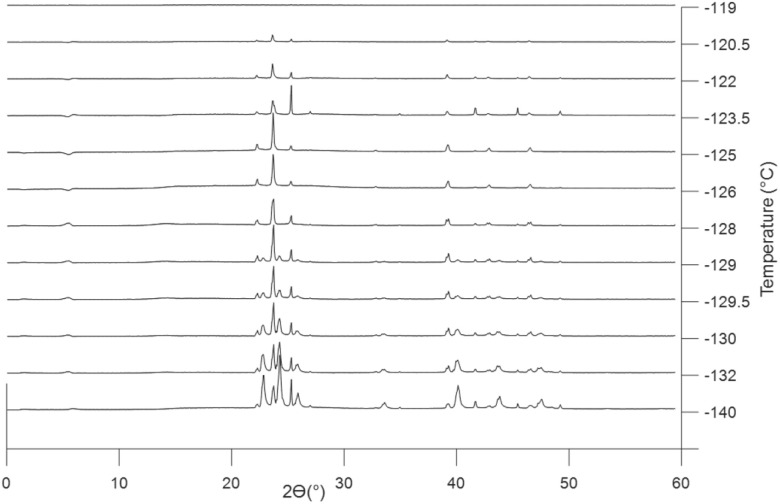
Crystal phase transition of confined [BMIM][Cl] and completely molten at −119 °C. The GISAXS pattern of confined [BMIM][Cl] at 25 °C is subtracted as the background. The vertical axis represents integrated intensity (a.u.).

Phase behavior was examined for [BMIM][Cl] confined in silica nanoporous films with both 2.3 and 8.2 nm diameter pores, with and without pore surface tethering. Surprisingly, for [BMIM][Cl], crystallization was only observed when confined in bare (non-tethered) 8.2 nm silica channels. For 2.3 nm bare pores and either pore size with IL tethering, no crystallization was observed during cooling or heating, or upon aging at room temperature, suggesting that the crystalline phases of [BMIM][Cl] were destabilized by the presence of pores, even if layering of ions near the pore surface is expected due to nanoconfinement. The GIWAXS results for 8.2 nm silica pores (Fig. 10) show a mixture of two crystal phases at the lowest temperature, −140 °C. Upon heating, one of the crystal phases melted around −128 °C. The remaining polymorph, crystal II, melted completely at −119 °C, which is much lower than for unconfined [BMIM][Cl]. The quantitative analysis combined with crystal structure fitting starting from the literature^45^ performed using TOPAS software gives a composition of about 68.6% of crystal I and 34.4% of crystal II at −140 °C. The descending fraction of crystal I is apparent in Fig. 10 and the structure is determined to be a monoclinic phase in crystal II. No nanodomains were observed in molten confined [BMIM][Cl] in any of the four cases, which is the same response as the nanodomains in [BMIM][PF_6_].

### Surface energy *vs.* surface-induced ordering

**Fig. 11:**
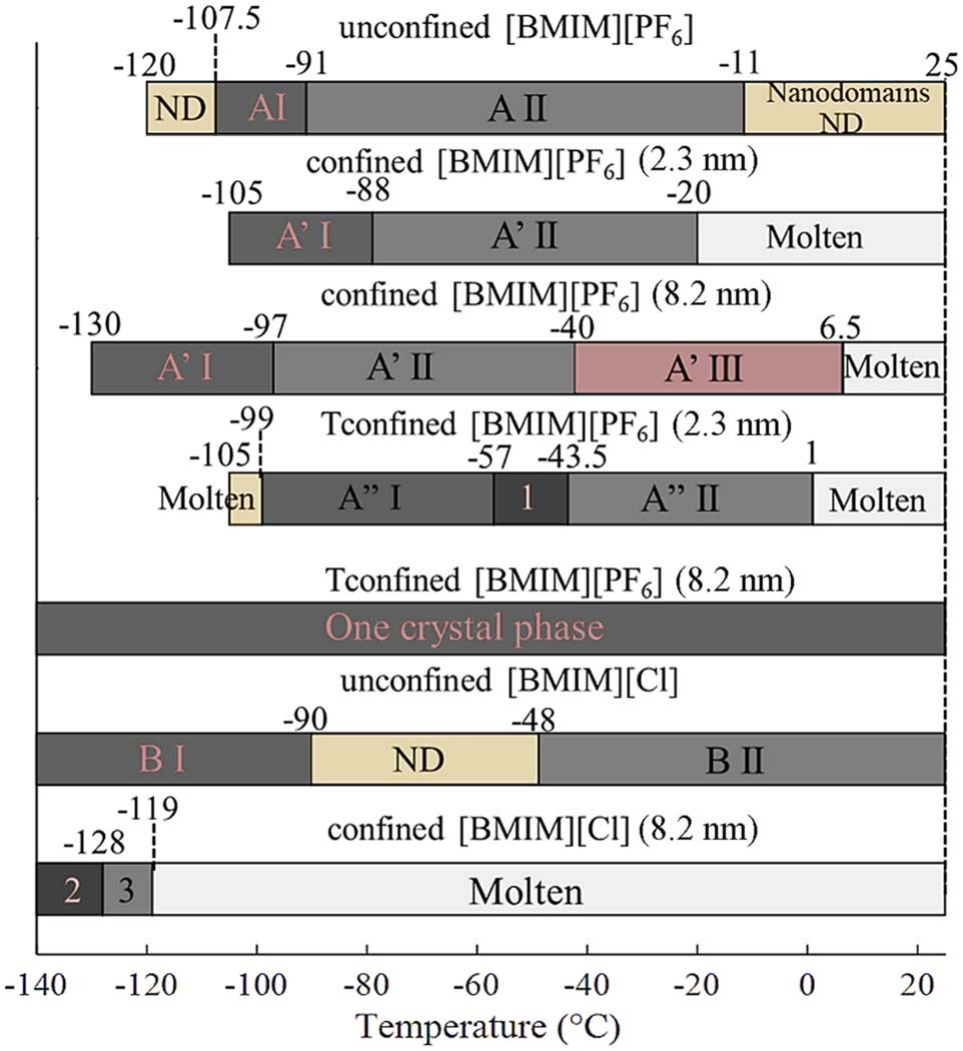
Crystal phase transition temperatures of A: unconfined [BMIM][PF_6_], B: unconfined [BMIM][Cl], A′: confined [BMIM][PF_6_], B′: confined [BMIM][Cl], and A′′: confined [BMIM][PF_6_] in [TMS-MIM][Cl] tethered pores. I and II represent the first and second crystal phases that appeared in the corresponding samples. The notations are 1 = A′′I + A′′II; 2 = B′I + B′II; 3 = B′II′.

By analogy with prior studies of both [BMIM][PF_6_] and [BMIM][Cl], the observation of different crystal polymorphs in this study can be attributed to different conformations of the butyl chain in [BMIM]^+^.^45,69,74,100^ The crystal phase transition temperatures of all samples showing crystallization are summarized and compared in Fig. 11. The corresponding unit cell parameters from TOPAS fitting are tabulated in Table 1. Details of the fitting are provided in the SI. All of the patterns were fitted well using crystal structures for bulk [BMIM][PF_6_] and [BMIM][Cl] reported in the literature^41^ with refined unit cell parameters and space groups. Because of a lack of isotropic crystals, structural modeling based on the WAXS patterns was not possible as it was with large single crystals of [BMIM][PF_6_].^101^ The crystal densities are in general smaller for the crystal phases at a higher temperature in the same sample. This is caused by the tendency for thermal expansion with increasing temperature.

**Table 1:** Unit cell parameter refinement results BMIM][PF_6_] of crystal phases^a^ identified in Fig. 11

	Space group	*a* (Å)	*b* (Å)	*c* (Å)	*α* (°)	*β* (°)	*γ* (°)	Density (g cm^−3^)
**[BMIM][PF**_**6**_ **]**								
**Unconfined**								
A I −103 °C	*P*1̄	9.08	7.67	5.90	102.51	115.23	109.23	2.96
A II −88 °C	*P*1̄	8.21	8.96	8.89	95.76	118.51	103.24	1.74
**Bare 2.3 nm silica confinement**								
A′I −91 °C	*P*1̄	8.00	11.81	8.89	86.48	94.67	92.55	1.13
A′II −84 °C	*P*1̄	7.11	8.85	13.37	91.67	114.80	106.15	1.31
**Bare 8.2 nm silica confinement**								
A′I −130 °C	*P*1̄	7.77	8.89	8.49	96.84	114.38	102.27	1.86
A′II −61 °C	*P*1̄	7.56	9.22	9.00	97.12	114.85	102.61	1.75
A′III 0.5 °C	*P*1̄	8.63	8.79	9.03	95.67	114.38	103.24	1.59
**Tethered 2.3 nm silica confinement**								
A′′I −49.5 °C	*P*1̄	9.02	9.48	9.05	94.64	113.68	101.83	1.38
A′′II −9.5 °C	*P*1̄	8.73	8.97	8.94	95.77	115.30	102.95	1.57
**Tethered 8.2 nm silica confinement**								
A′′ 25 °C	*P*1̄	8.61	9.13	9.02	94.60	116.39	102.85	1.56

**[BMIM][Cl]**								
**Unconfined**								
BI −120 °C	*P*12_1_/*c*1	11.44	10.05	8.87	90.00	115.21	90.00	1.26
BII 39 °C	*P*12_1_/*c*1	9.87	11.95	9.73	90.00	119.89	90.00	1.17
**Bare 8.2 nm silica confinement**								
B′I −140 °C (68.55%)	*C*2	9.39	16.62	8.51	90.00	124.63	90.00	1.06
B′II −140 °C (31.45%)	*P*12_1_/*c*1	9.85	12.22	9.76	90.00	120.07	90.00	1.14

a Crystallography information from Choudhury *et al.*^41,45^ ([BMIM][PF_6_]) and Holbrey *et al.*^45^ ([BMIM][Cl]). A/B: unconfined IL. A′/B′: confined IL. A′′: Tconfined IL.

The first general trend found in Fig. 11 is that GIWAXS indicates that nanodomains found in the bulk molten state for both [BMIM][PF_6_] and [BMIM][Cl] are eliminated by nanoconfinement. These nanodomains represent ordered small clusters of IL at equilibrium with disordered liquid, so it is likely that liquid layering at the pore surface lowers the driving force for the formation of ordered nanodomains in the fluid inside the pore. The nanodomains are associated with cold crystallization (crystallization during heating under severe subcooling). Since the nanodomains are not present to stabilize the liquid phase, cold crystallization is not found in fluids confined in bare silica pores. Also, the observation of melting point elevation in some cases for [BMIM][PF_6_] suggests that nanoconfinement-induced layering plays an important role in the behavior of ILs and either contributes to or completely opposes the melting point depression usually expected according to the Gibbs–Thomson effect. This is most exaggerated for [BMIM][PF_6_] in 8.2 nm IL-tethered pores, where nucleation is slow but a single crystalline phase forms that is stable up to room temperature. In contrast to these effects, strong and specific interactions between BMIM^+^ and Cl^−^, which are responsible for the high melting temperature of pure [BMIM][Cl], are disrupted by layering at the pore surface in the liquid state. This leads to severe melting point depression (observed only in 8.2 nm bare silica pores) or suppression (observed in smaller silica pores and with IL tethering).

The large depression or even suppression of melting of [BMIM][Cl] in silica nanopores found in this study suggests that the impacts of confinement on crystallization of [BMIM][Cl] are much greater than for [BMIM][PF_6_]. The different melting point changes of [BMIM][Cl] and [BMIM][PF_6_] in response to bare and tethered silica nanoconfinement are most likely due to the stronger H-bonding between BMIM^+^ and Cl^−^.^45,95^ A previous FTIR study by He *et al.* shows evidence for an interaction between silica pore walls and [BMIM]^+^ in [BMIM][Cl].^59^ When the relatively strong interaction between [BMIM]^+^ and Cl^−^ is disturbed, the lack of driving force for specific molecular orientation in a crystal might cause the large depression or suppression of *T*_m_ of confined [BMIM][Cl] compared to bulk. With most of the confined [BMIM][Cl] displaying *T*_m_ suppression, it is difficult to compare and model different confinement environments on [BMIM][Cl], but conditions explored here all result in [BMIM][Cl] with much lower *T*_m_, which suggests that when nanoconfined, it can be used over a wide temperature range as a true ionic liquid.

## Conclusions

Temperature-resolved *in situ* GIWAXS was successfully performed to probe the crystallization of unconfined [BMIM][PF_6_] and [BMIM][Cl], as well as the two ILs in 2.3 nm and 8.2 nm-pore-diameter nanoporous silica films with and without [TMS-MIM][Cl] tethering. Short-range nanodomains were observed in both unconfined ILs even when they were in the amorphous state. This experimental evidence agrees with computational studies suggesting the presence of heterogeneous ordered clusters coexisting with disordered liquid in bulk ILs. When [BMIM][PF_6_] was confined in 2.3 nm silica nanopores, it exhibited *T*_m_ depression by 9 °C as predicted using the Gibbs–Thomson equation. However, the Gibbs–Thomson effect is not applicable when it comes to [BMIM][PF_6_] confined in 8.2 nm pores where *T*_m_ is elevated by 17.5 °C. This unconventional behavior is attributed to more space in the 8.2 nm pores allowing [BMIM][PF_6_] to layer more extensively, which stabilizes the crystalline IL. When the silica pore surface was tethered with [TMS-MIM][Cl], confined [BMIM][PF_6_] in both sizes of pore showed higher *T*_m_ because the pore surface is more like the IL itself and no effects due to hydrogen bonding with SiOH (or electrostatic interactions with SiO^−^) groups are seen. These effects are expressed most strongly in 8.2 nm pores where layering and tethering favor crystallization. [BMIM][PF_6_] presented only one stable crystal phase under this condition that did not melt at room temperature. This provides a new method of stabilizing the complicated crystallization behavior of [BMIM][PF_6_] and suppressing polymorphs, which gives better control of crystal phases of [BMIM][PF_6_] over a wider temperature range.

The other IL studied, [BMIM][Cl], has a smaller anion that interacts more strongly and specifically with the cations. For the conditions explored here, its crystallization under nanoconfinement was found to occur only in 8.2 nm bare silica pores, but with an unusually large *T*_m_ depression of −119 °C. This is suspected to result from the disturbance of the cation–anion interaction in the crystalline state, which is the primary driving force for stable ordering of [BMIM][Cl]. Simulations suggest that alternating ion layering still happens over a large distance in the liquid state, but in this case layering lowers the ability of the system to assemble into crystalline solids. Because [BMIM][Cl] is found in the liquid form over a much wider temperature range than in the bulk fluid, nanoconfinement should enhance its properties for uses requiring a liquid form below the bulk melting point. The findings from this study give some guidance to the range of crystallization behaviors that can be found for ionic liquids by tailoring the pore size and surface properties depending on the behavior of the bulk IL. By varying the crystal phase transition temperatures, nanoconfinement creates new opportunities for applications of ILs in fields including drug delivery, separations, electrochemical devices and catalyst supports.

## Author contributions

Y. H., M. A. K., J. S., F. L., B. L. K. and S. E. R. designed the research; Y. H., M. A. K., A. D. D., J. G., A. S. and S. G. performed the research; Y. H., J. S. and S. E. R. analyzed the data; Y. H., J. S., F. L., B. L. K. and S. E. R. wrote the paper.

## Conflicts of interest

There are no conflicts to declare.

## Abbreviations

BMIM: 1-Butyl-3-methylimidazolium
CTAB: Cetyltrimethylammonium bromide
DSC: Differential scanning calorimetry
GISAXS: Grazing incidence small angle X-ray scattering
GIWAXS: Grazing incidence wide angle X-ray scattering
IL: Ionic liquid
RTD: Resistance temperature detector
SIL: Supported ionic liquid
Tconfined: Pore tethered and confined
*T*_m_
: Melting temperature
TMS-MIM: 1-(3-Trimethoxysilylpropyl)3-methylimidazolium
XPS: X-ray photoelectron spectroscopy

## Supplementary Material

NA-007-D5NA00509D-s001

## Data Availability

Data for this article, including raw GISAXS and GIWAXS datasets and processed Excel files for the figures presented in this manuscript, are available at the Open Science Framework at https://doi.org/10.17605/OSF.IO/Z846G. Supplementary information available: a summary of [BMIM][PF_6_] melting temperatures reported in the literature; methods of thin film synthesis and characterization; the temperature calibration procedure and results for the GIWAXS stage; XPS etching results for P123 and CTAB templated thin films; the GIWAXS pattern of silicon wafer plus Kapton background; GIWAXS and GISAXS patterns of the CTAB templated film; raw GIWAXS patterns of [BMIM][PF_6_] in silica films with 2.3 nm pores; and crystal structure analyses for representative GIWAXS patterns. See DOI: https://doi.org/10.1039/d5na00509d.
