# Supplementary material for: Crystallization of nanopore-confined imidazolium ionic liquids probed by temperature-resolved in situ grazing-incidence wide angle X-ray scattering (GIWAXS)
Source: Nanoscale Adv. 2025 Jul 28;7(20):6607–19. doi: 10.1039/d5na00509d (PMC12415534; doi:10.1039/d5na00509d)
Supplement: NA-007-D5NA00509D-s001 [file NA-007-D5NA00509D-s001.pdf]

Supplementary Information (SI) for Nanoscale Advances.
This journal is © The Royal Society of Chemistry 2025

Supplementary Information

Crystallization of Nanopore-Confined Imidazolium Ionic Liquids Probed by Temperature-resolved *In Situ* Grazing-Incidence Wide Angle X-ray Scattering (GIWAXS)

Yuxin He, Arif M. Khan, Andrew D. Drake, Joshua Garay, Aniruddha Shirodkar, Stephen Goodlett, Joseph Strzalka, Folami Ladipo, Barbara L. Knutson and Stephen E. Rankin**

Y. He, M.A. Khan, A.D. Drake, J. Garay, A. Shirodkar, B.L. Knutson, and S.E. Rankin
Department of Chemical and Materials Engineering
University of Kentucky
177 F.P. Anderson Tower
Lexington, Kentucky, 40506-0046, USA
E-mail: bknut2@uky.edu (B.L.K.) and stephen.rankin@uky.edu (S.E.R.)

J. Strzalka
X-ray Science Division
Argonne National Laboratory
Argonne, Illinois, 60439, USA

S. Goodlett, F. Ladipo
Department of Chemistry
University of Kentucky
125 Chemistry/Physics Building
Lexington, Kentucky, 40506-0055, USA

Table S1. Summary of T_m values of bulk [BMIM][PF_6] reported in previous studies.

Method	Melting Point	Reference
Cooled at > 0.02 K/s below -63 °C then slow heating	11 °C	[1]
Heated from -100 °C	10 °C	[2]
Cooled to -150 °C at 6 °C/min then heating at 10 °C/min	1.9 °C	[3]
Cooled below -100 °C then heating at 10 °C/min	6.6 °C	[4]
Heating from -90 °C to 40 °C at 0.3 °C/min	11 °C	[5]
Not specified	4 °C	[6]
Cooled to -30 °C followed by shock-induced crystallization	11 °C	[7]

Experimental Methods for Film Synthesis and Functionalization

Materials. NoChromix power, titanium (IV) isopropoxide (TIP, ⩾97%), glycerol (89%), 1,6-diisocyanatohexane (98%), tetraethoxysilane (99.9%), and Pluronic P123 poly(ethylene oxice)-b-poly(propylene oxide)-b-poly-(ethylene oxide) copolymer (M_n ~ 5800) were obtained from Sigma-Aldrich; deionized ultrafilterd (DIUF) water from Fisher Scientific; concentrated sulfuric acid (98%), 1 N HCl, and acetone (99.5%) from VWR; cetyltrimethylammonium bromide (CTAB, 99.8%) from MP Biomedicals; tetraethyl orthosilicate (TEOS, 99%) from Acros Organics; sugar surfactant n-dodecyl β-D-maltopyranoside ($C_{12}G_2$, HPLC grade, ⩾99%) from BioVision Inc.; and 200 proof ethanol from Decon Laboratories. The substrates used for sample preparation were borosilicate glass slides (VWR) and 600 μm thick silicon (Si) wafers (University Wafer, Inc.). Kapton film (125 μm) was purchased from The McCrone Group, Inc.

Pluronic P123 templated silica film preparation: Silica films with hexagonal close packed pores oriented orthogonally to the substrates are prepared by using the sol-gel method of Koganti and Rankin.[8] The silicon (Si) wafer substrates were cut into approximately 1 cm x 3 cm pieces cleaned with NoChromix in sulfuric acid, rinsed with DIUF water. After cleaning, Si wafers were modified with Pluronic surfactant P123 crosslinked using 1,6-diisocyanatohexane (0.696 mM of each reagent in acetone with a drop of glycerol for crosslinking). The Si wafers were then dip coated with silica precursor solution with a final molar ratio of tetraethoxysilane (TEOS): ethanol: DIUF water: HCl: P123 of 1: 22: 5: 0.0004: 0.01. Finally, the silica films were cured, and the modifying layer and templating surfactant were removed by calcinating at 500 °C for 4 h in air. More details can be found in He et al. [9]

Cetyltrimethylammonium bromide (CTAB) templated silica thin film preparation: CTAB was used as surfactant to synthesize silica thin films with nanochannels perpendicular to the substrate with diameter of about 2.3 nm. Silica thin films with 2% titania were synthesized by mixed templating using CTAB and a sugar-based surfactant ($C_{12}G_2$) which complexes with the Ti-precursor, following a modified procedure of Rahman et al. [10] Initially, 70 mg of $C_{12}G_2$, was dried in a vacuum oven at 50 °C for 24 h and then dissolved in 2.4 mL of dry ethanol. 42 µL of TIP was added to this ethanolic solution (resulting in a Ti:$C_{12}G_2$ molar ratio of = 1:1) and the solution was stirred for 3 h in a sealed vial to allow for the complexation of TIP with the sugar headgroup of $C_{12}G_2$. To prevent contact with moisture in the air, the procedures above were conducted in a glove bag filled with dry nitrogen. Separately, 1.41 mL of TEOS was added to 0.98 mL ethanol under constant stirring and 0.21 mL of DIUF water and 0.27 mL of 0.1 M HCl was added to this solution. The TEOS mixture was stirred for an hour to allow for the hydrolysis of silica precursor. The TEOS solution was then added dropwise to the TIP:$C_{12}G_2$ mixture with

vigorous mixing in a nitrogen glove bag. The mixture was then stirred while an additional 0.12 mL of DIUF water and 1.03 mL of ethanol were added. Finally, 252 mg of CTAB was added to the reaction mixture followed by 3.42 mL ethanol. This solution was stirred for an hour before dip coating.

To promote the formation of a vertically oriented pore structure, CTAB-templated thin films were coated onto borosilicate glass slides. The glass slides were cleaned with NoChromix in concentrated sulfric acid solution then rinsed with DIUF water and blow dried. The cleaned glass slides were coated with the titanium-doped silica sol (withdrawal speed 6 cm/min) and then placed into an oven to age at 50 °C for 48 h. Aged films were heated at 120 °C for 6 h and then calcined in air at 500 °C for 1 h with a heating rate of 1 °C/min.

1-(3-trimethoxysilylpropyl)3-methylimidazolium chloride [TMS-MIM][Cl] tethering: The imidazolium-based silane [TMS-MIM][Cl] was synthesized by reacting N-methylimidazole (2.00 ml, 25 mmol) and (3-chloropropyl)trimethoxysilane (4.59 ml, 25 mmol) under reflux in toluene (40 ml) for 36 h. The mixture was allowed to cool to room temperature and the supernatant was removed with a pipette. The resultant oil was washed with diethyl ether (10 ml for three times) to remove unreacted materials, and then dried under reduced pressure at room temperature. The product was obtained as a colorless oil (6.80 g, 97%). The identity of the prepared IL-like organosilane was established by comparing its ^{1}H and ^{13}C NMR spectra with the reported literature.[11] Both solvents, toluene and diethyl ether dried and distilled by standard methods prior to use.[12] For IL tethering, silica films obtained after calcination were submerged in a solution of 0.05 mol/L [TMS-MIM][Cl] in chloroform. This step was carried out in a nitrogen glove bag to prevent [TMS-MIM][Cl] from being hydrolyzed by moisture. The

submerged film was heated at 60 °C for 24 h under reflux. After that, the silica films were removed from the solution and rinsed thoroughly with ethanol and DI water.

Temperature Calibration for GIWAXS Stage

For *in situ* temperature-controlled GIWAXS measurements, temperature was measured using a thermocouple (**Scheme S1**). However, the temperature at the surface of the substrate (where the silica films and ionic liquid were located) needed to be calibrated due to the finite thermal conductivity of glass and silicon wafers. The procedures of measuring the heating and cooling curves on the sample surface with an external thermostat are described in the Experimental section of the main text.

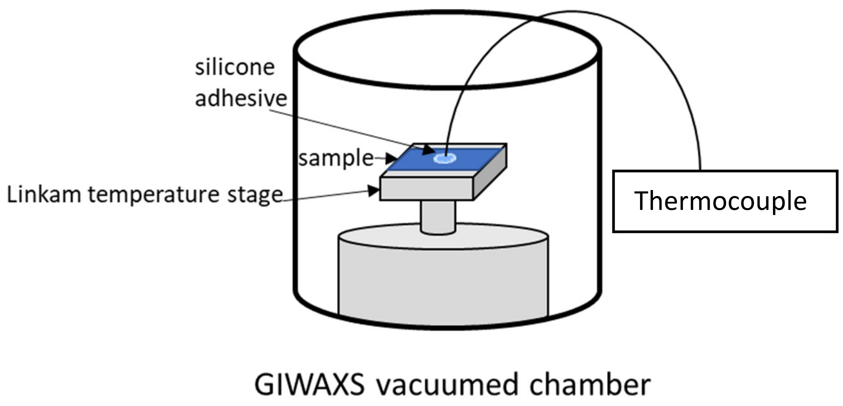

Scheme S1. Sample surface temperature measurement with an external thermostat

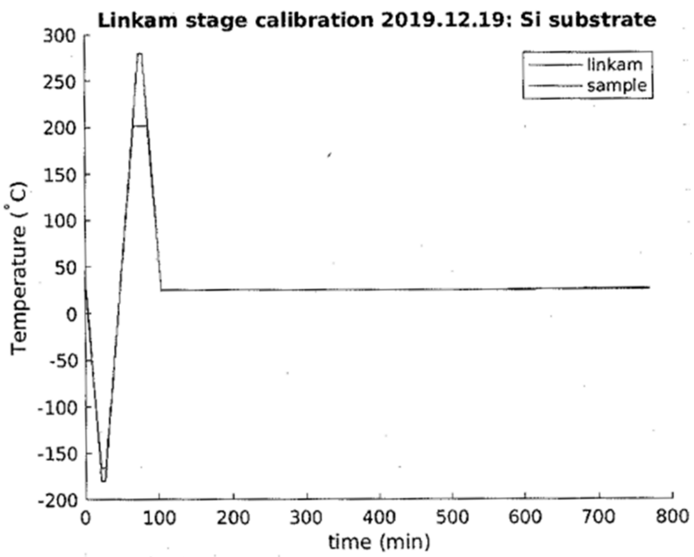

Figure S1. Temperature curves with respect to time from Linkam stage and external thermostat (sample) temperature reading of a silica film deposited onto a Si wafer. The temperature at the top of the film follows the base plate temperature closely except at the maximum temperature.

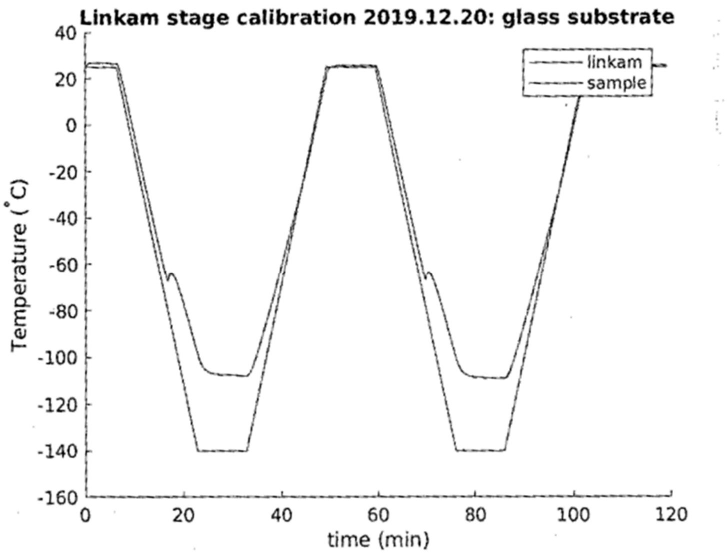

Figure S2. Temperature curves with respect to time from Linkam stage and external thermostat (sample) temperature reading of silica film deposited onto a glass slide. Note the offset at low temperature.

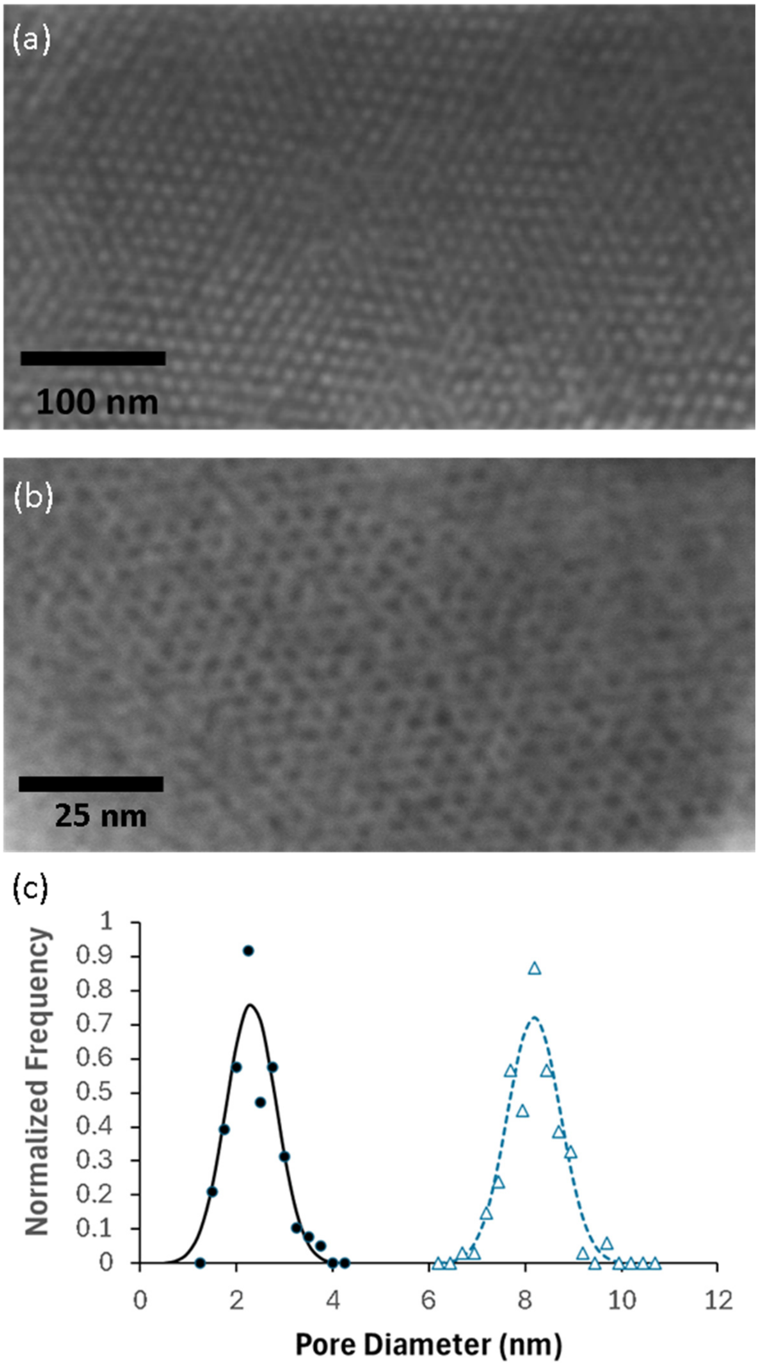

Figure S3. (a) Brightfield TEM of P123-templated silica film with vertically oriented channels after calcination, (b) darkfield scanning TEM image of mesoporous silica film doped with 3 mol% TiO_2, pores templated with CTAB and calcined in air at 500 °C, and (c) pore size distributions derived from the images using thresholding and particle analysis tools in ImageJ software, giving means and standard deviations of 2.3 ± 0.5 nm (n=141) for the CTAB film (filled circles) and 8.2 ± 0.5 (n=150) for the P123 film (open triangles). Image (a) was collected using a JEOL 2010F microscope, and image (b) using a FEI Talos F200X microscope, both at 200 kV acceleration voltage.

Table S2. Atomic percentages of N, O, Si, and O in P123 templated (8.2 nm pore) silica nanoporous film with [TMS-MIM][Cl] tethering as a function of cumulative etch time from a XPS depth profile. Si wafer used as the substrate.

Etch Time (s)	Etch Level	Elements Atomic %			
		**N1s**	**O1s**	**Si2p**	**C1s**
0	0	2.03	58.5	29.3	10.2
10	1	1.89	61.0	31.7	5.43
20	2	1.79	60.7	31.7	5.83
30	3	2.07	60.2	31.5	6.27
40	4	2.13	59.5	31.7	6.71
240	5	2.37	58.7	32.4	6.53
440	6	2.16	60.2	32.1	5.51
640	7	0.00	4.53	95.5	0.00
840	8	0.00	3.89	96.1	0.00
1040	9	0.00	3.03	97.0	0.00
1240	10	0.00	3.50	96.5	0.00
1440	11	0.00	3.42	96.6	0.00
1640	12	0.00	3.42	96.6	0.00

Table S3. Atomic percentages of N, O, Si, and O in CTAB templated (2.3 nm pore) silica nanoporous film with [TMS-MIM][Cl] tethering as a function of cumulative etch time from a XPS depth profile. A glass slide was used as the substrate.

Etch Time (s)	Etch Level	Elements Atomic %			
		**N1s**	**O1s**	**Si2p**	**C1s**
0	0	4.38	50.4	24.6	20.6
10	1	3.00	56.6	29.6	10.8
20	2	3.22	56.1	29.6	11.1
30	3	3.36	56.0	29.4	11.2
40	4	3.45	56.1	29.4	11.1
240	5	3.58	55.1	29.5	11.8
440	6	0.00	67.4	31.9	0.69
640	7	0.00	67.5	32.0	0.53
840	8	0.00	67.7	31.9	0.47
1040	9	0.00	67.6	32.4	0.00
1240	10	0.00	67.3	32.3	0.48
1440	11	0.00	67.2	32.3	0.46
1640	12	0.00	67.2	32.3	0.43

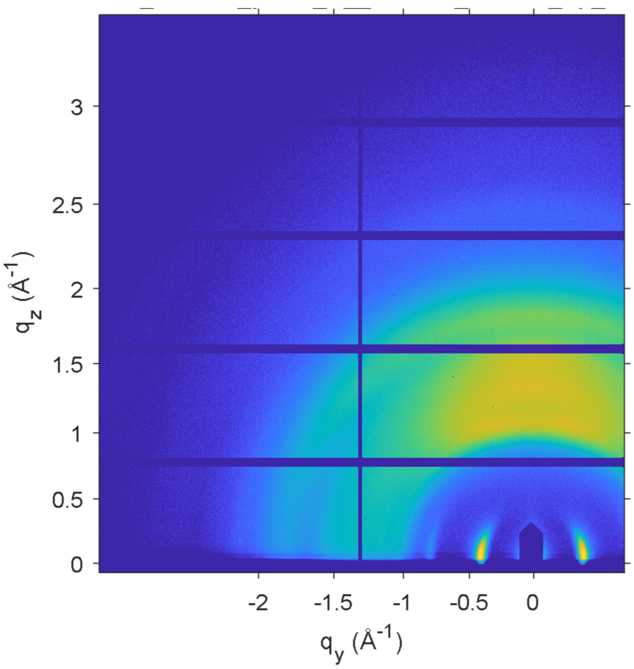

Figure S4. GIWAXS pattern of Si wafer and Kapton film background.

X-ray Scattering Background Patterns

Figure S4 shows the GIWAXS background from the Si wafer and Kapton film. This pattern was used for background subtraction during processing of confined IL films. The GIWAXS pattern at low scattering vector of IL confined in mesoporous films were also be used to confirm the presence of the vertical pore structure with IL loading at low temperature. While the focus in the main text is the evolution of crystal structure, the expansion of the GIWAXS pattern (**Figure S5a**) at low q values (**Figure S5b**) yields a pattern that matches what is observed at better resolution and without an interfering beam stop by GISAXS (**Figure S5c**). The vertical rods found to the left and right of the beam stop confirm a vertically aligned channel pore structure.

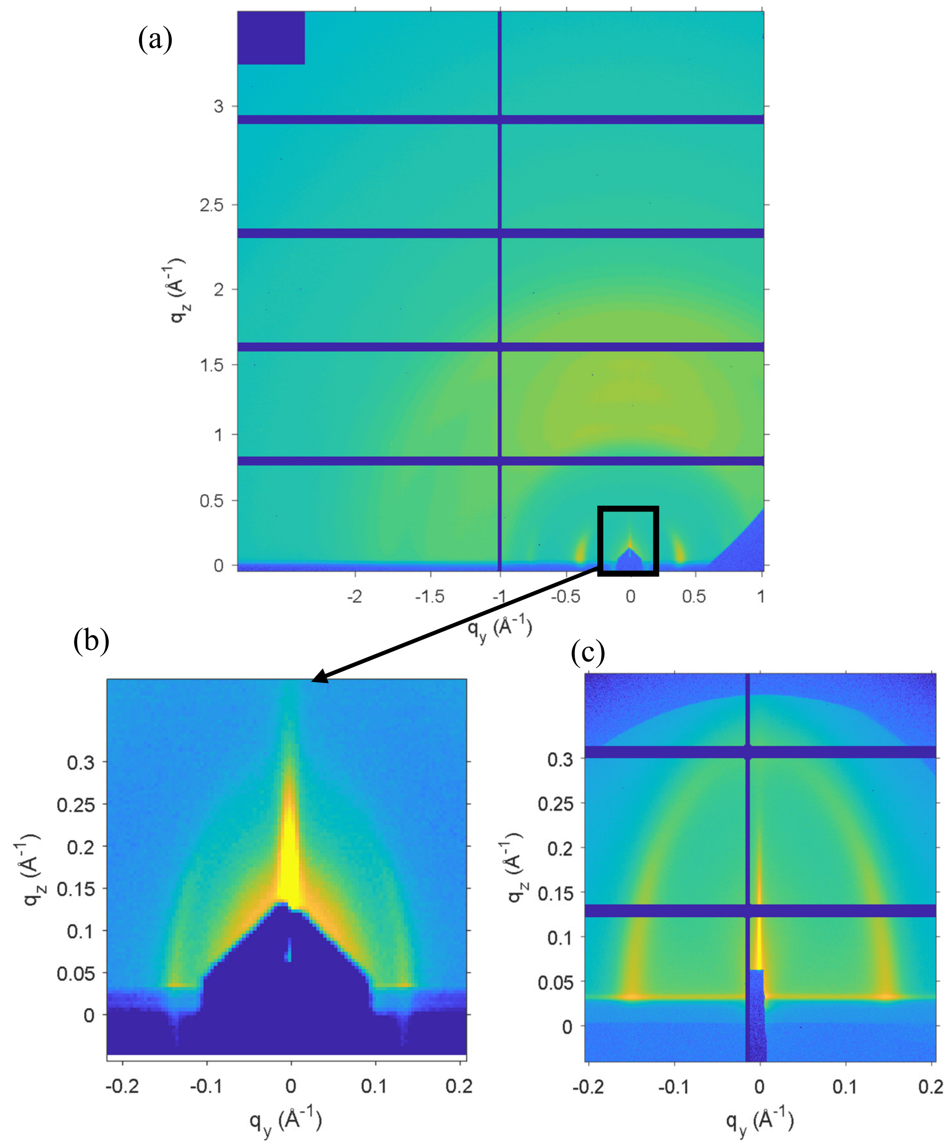

Figure S5. GIWAXS patterns of (a) [BMIM][PF_6] confined in 2.3 nm porous silica film at -103.5 °C; (b) low q region of pattern (a); and (c) GISAXS patterns of CTAB templated silica film with 2.3 nm pores.

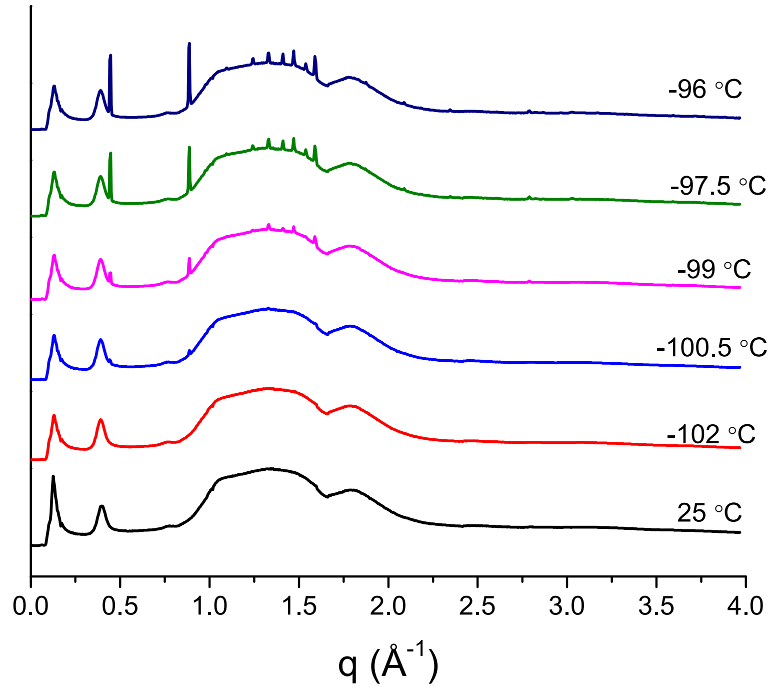

Figure S6. GIWAXS patterns of [BMIM][PF_6] confined in tethered 2.3 nm porous silica thin films from -102 °C to -96 °C with the pattern at 25 °C (melted) at the bottom showing the background with no crystallization

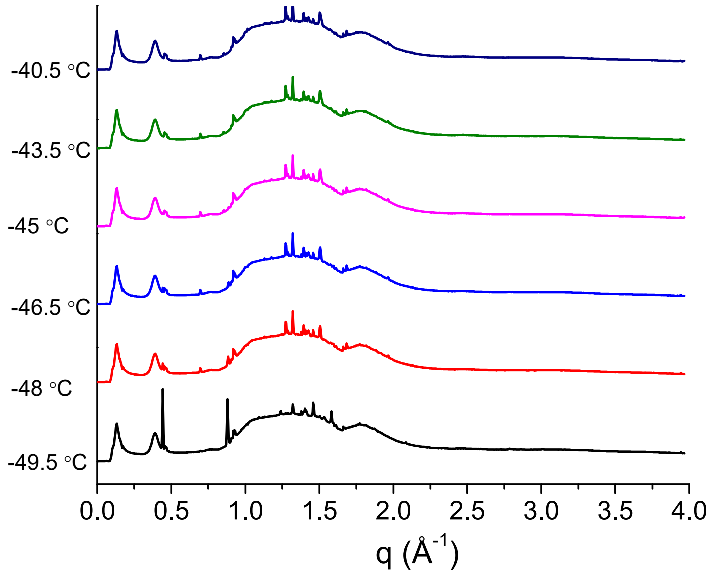

Figure S7. GIWAXS patterns of [BMIM][PF_6] confined in tethered 2.3 nm porous silica thin films from -49.5 °C to -40.5 °C showing the complete disappearance of the phase I with the two intense peaks around 0.4 and 0.8 $Å^{-1}$ at -43.5 °C

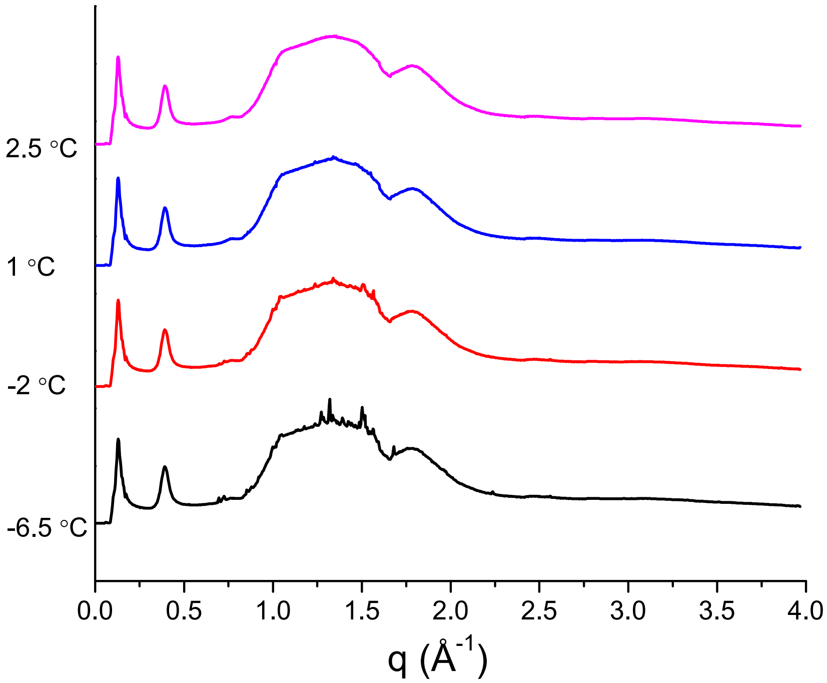

Figure S8. GIWAXS patterns of [BMIM][PF_6] confined in tethered 2.3 nm porous silica thin films from -6.5 °C to 2.5 °C showing the complete melting of phase II at 1 °C

Crystal Structure Analysis

2D GIWAXS patterns of each samples are shown below followed by TOPAS crystallography structure refinement output files. The atomic coordinates, atom occupancy and equivalent temperature factor (B_{eq}) are fixed, so they are only listed once for both ILs.

Because of the unconventional thin film form of sample for GIWAXS measurements of nanoconfined ILs, which involve heterogeneous soft and hard materials, there are greater variations in peak intensities compared to powder patterns since some of the rings from GIWAXS patterns are not complete. Thus, during crystal refinement, greater R-values (about 30) are allowed compared to the common suggested value of 20. The 2θ values are converted from q (Å^{-1}) using a wavelength of 1.54 Å (Cu Kα) for easier refinement from literature crystal structure information in terms of angle. The unit cell parameters resulting from this analysis are summarized in the main text.

Unconfined [BMIM][PF_6] at -103 °C

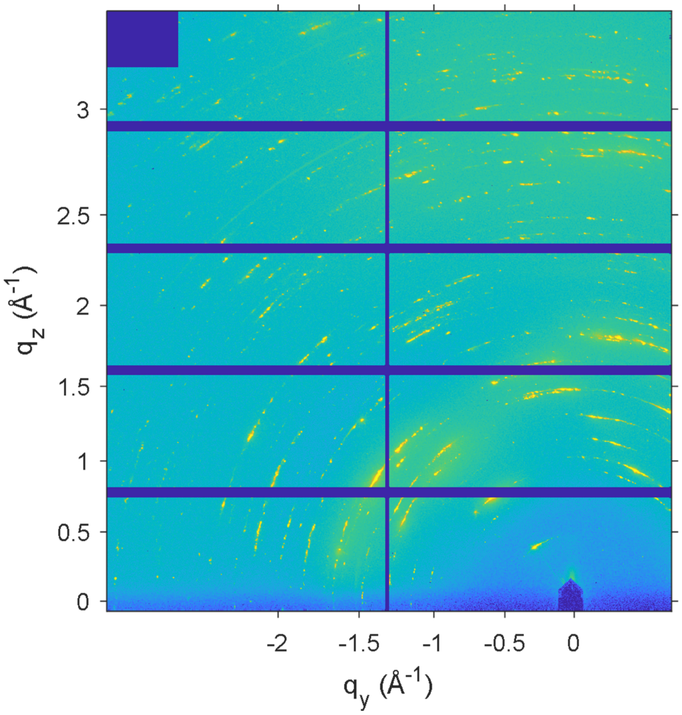

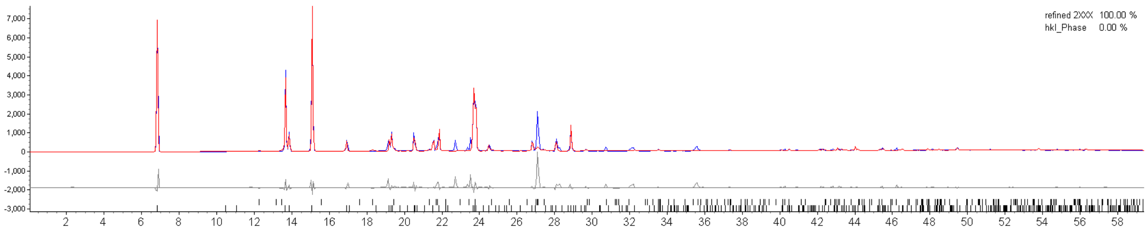

R-Values

```
Rexp : 7.19     Rwp : 29.69     Rp  : 18.68   GOF : 4.13
Rexp`: 10.36    Rwp`: 42.78     Rp` : 33.04   DW  : 1.03
```

Quantitative Analysis - Rietveld

```
   Phase 1  : "refined 2XXX"                 100.000 %
   Phase 2  : hkl_Phase                      0.000 %
```

Background

```
   Chebychev polynomial, Coefficient  0      54.00018
                                      1      49.34894
                                      2      -3.995787
                                      3      -0.568013
                                      4      -3.262048
                                      5      -3.763277
```

Instrument

```
   Primary radius (mm)                       217.5
```

Secondary radius (mm) 217.5

Corrections

Zero error	0.01670187
LP Factor	0

Structure 1

Phase name	refined 2XXX
R-Bragg	45.930
Spacegroup	P-1
Scale	3.69059398e-005
Cell Mass	568.372
Cell Volume (Å^3)	318.63200
Wt% - Rietveld	100.000
Crystallite Size	
Cry size Lorentzian (nm)	58.9
Crystal Linear Absorption Coeff. (1/cm)	50.036
Crystal Density (g/cm^3)	2.962
PVII peak type	
FWHM = a + b/Cos(Th) + c Tan(Th)	
a	0.01417506
b	0.007878899
c	0.0001
Exponent m = 0.6+ma+mb/Cos(Th)+mc/Tan(Th)	
ma	19.99996
mb	4.999994
mc	4.999981
Lattice parameters	
a (Å)	9.0784892
b (Å)	7.6703595
c (Å)	5.8971551
alpha (°)	102.5097
beta (°)	115.2332
gamma (°)	109.2292

Site	Np	x	y	z	Atom	Occ	Beq
P1	2	0.18444	0.79488	0.44901	P	1	2.199
F1	2	0.35521	0.93965	0.57091	F	1	3.237
F2	2	0.29779	0.67516	0.50853	F	1	3.869
F3	2	0.12957	0.78229	0.59432	F	1	4.256
F4	2	0.07314	0.91488	0.38757	F	1	3.664
F5	2	0.24066	0.81036	0.30242	F	1	4.295
F6	2	0.01489	0.65083	0.32406	F	1	4.035
N1	2	0.75199	0.72952	0.65019	N	1	2.132
N2	2	0.78894	0.83706	0.89274	N	1	2.187
C1	2	0.69310	0.69370	0.46853	C	1	3.182
H1A	2	0.58390	0.72160	0.41060	H	1	4.816
H1B	2	0.67150	0.58060	0.42940	H	1	4.816
H1C	2	0.78460	0.75510	0.44440	H	1	4.816
C2	2	0.70855	0.83227	0.72967	C	1	2.227
H2	2	0.63220	0.89270	0.67830	H	1	2.685
C3	2	0.86449	0.66566	0.76617	C	1	2.503
H3	2	0.91630	0.58900	0.74360	H	1	3
C4	2	0.88720	0.73249	0.91775	C	1	2.55
H4	2	0.95780	0.71150	1.02240	H	1	3.079
C5	2	0.76590	0.92927	1.02293	C	1	2.779

H5A	2	0.71160	1.01120	0.97740	H	1	3.316
H5B	2	0.88280	0.98340	1.11980	H	1	3.316
C6	2	0.65000	0.82430	1.08181	C	1	2.424
H6A	2	0.70700	0.74480	1.13030	H	1	2.921
H6B	2	0.64200	0.89010	1.17210	H	1	2.921
C7	2	0.46470	0.73904	0.94394	C	1	2.645
H7A	2	0.47170	0.66970	0.85540	H	1	3.158
H7B	2	0.40830	0.81790	0.89270	H	1	3.158
C8	2	0.35100	0.63970	1.00890	C	1	3.3
H8A	2	0.40610	0.56120	1.05940	H	1	4.974
H8B	2	0.23360	0.58560	0.91560	H	1	4.974
H8C	2	0.34000	0.70840	1.09360	H	1	4.974

Unconfined [BMIM][PF_6] at -88 °C

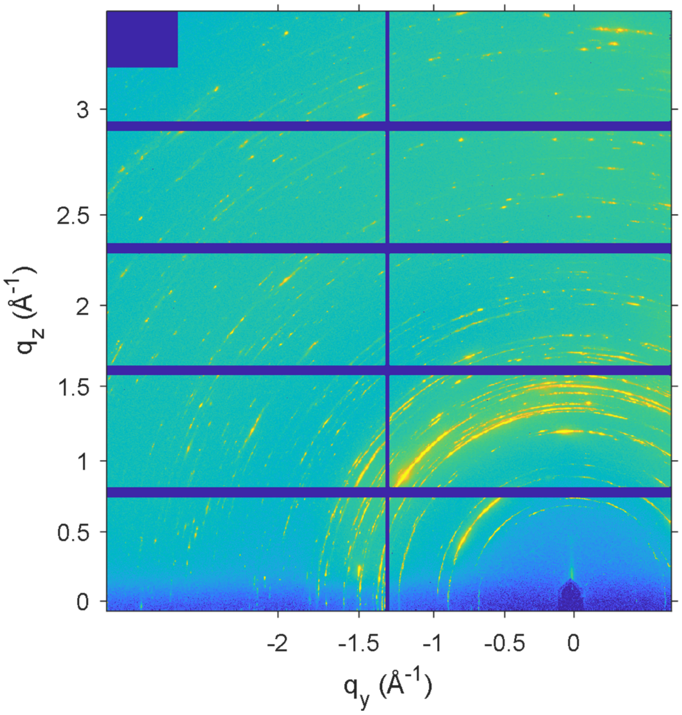

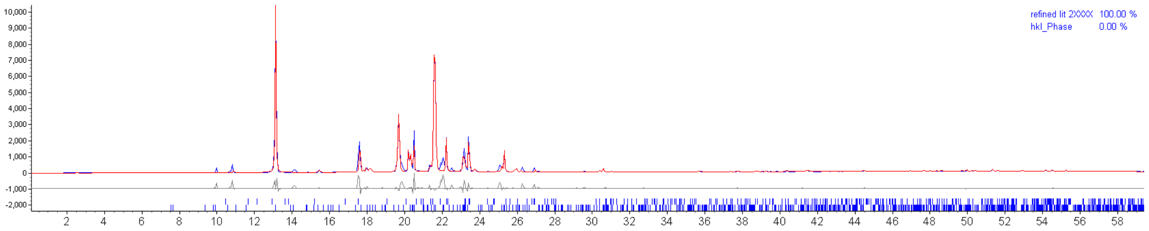

R-Values

```
Rexp : 3.23    Rwp : 25.19    Rp  : 14.10   GOF : 7.80
Rexp`: 4.40    Rwp`: 34.37    Rp` : 23.30   DW  : 1.34
```

Quantitative Analysis - Rietveld

```
   Phase 1  : "refined lit 2XXXX"           100.000 %
   Phase 2  : hkl_Phase                     0.000 %
```

Background

```
   Chebychev polynomial, Coefficient  0     56.69783
                                      1     56.44368
                                      2     -5.832474
                                      3     -10.62477
                                      4     -7.487998
                                      5     -3.686097
```

Instrument

```
   Primary radius (mm)                     217.5
   Secondary radius (mm)                   217.5
```

Corrections

Zero error	0.100127
Specimen displacement	9.842157
LP Factor	0

Structure 1

Phase name	refined lit 2XXXX
R-Bragg	21.148
Spacegroup	P-1
Scale	2.33069156e-006
Cell Mass	568.372
Cell Volume (Å^3)	543.06831
Wt% - Rietveld	100.000
Crystallite Size	
Cry size Lorentzian (nm)	9999.9
Crystal Linear Absorption Coeff. (1/cm)	29.357
Crystal Density (g/cm^3)	1.738
PVII peak type	
FWHM = a + b/Cos(Th) + c Tan(Th)	
a	0.3785677
b	0.0006103156
c	0.0001009526
Exponent m = 0.6+ma+mb/Cos(Th)+mc/Tan(Th)	
ma	19.99992
mb	4.999981
mc	4.999991
Lattice parameters	
a (Å)	8.2137512
b (Å)	8.9633556
c (Å)	8.8948718
alpha (°)	95.75617
beta (°)	118.5138
gamma (°)	103.2361

Confined [BMIM][PF_6] in 2.3 nm pores at -91 °C

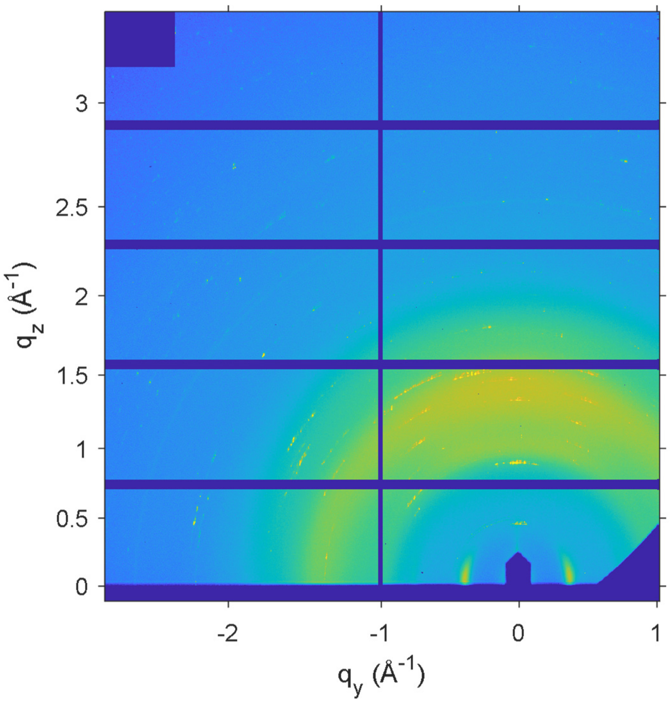

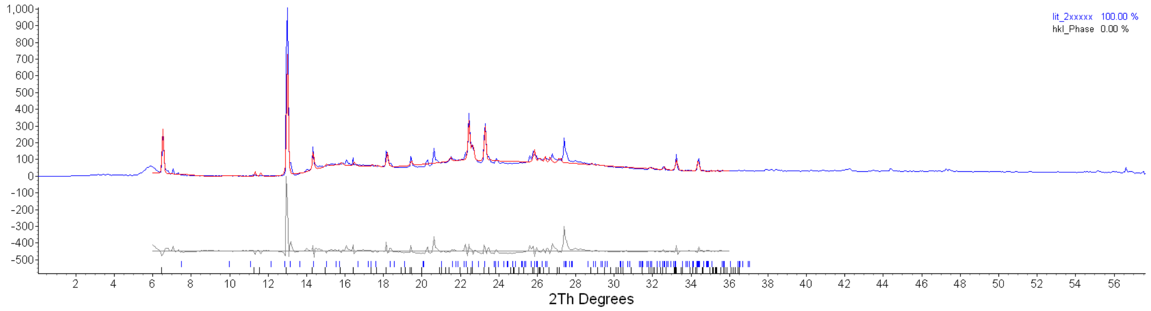

R-Values

```
Rexp : 11.03    Rwp : 23.08    Rp  : 15.21   GOF : 2.09
Rexp`: 11.38    Rwp`: 23.82    Rp` : 26.04   DW  : 1.02
```

Quantitative Analysis - Rietveld

```
   Phase 1  : lit_2xxxxx                     100.000 %
   Phase 2  : hkl_Phase                      0.000 %
```

Background

```
   Chebychev polynomial, Coefficient  0      20.15245
                                      1      22.74066
                                      2      -18.89273
                                      3      -21.22206
                                      4      12.59577
                                      5      1.905042
```

Instrument

Primary radius (mm)	217.5
Secondary radius (mm)	217.5

Corrections

Zero error	0.05894278
Specimen displacement	-0.02481741
LP Factor	0

Miscellaneous

Start X	6
Finish X	36

Structure 1

Phase name	lit_2xxxxx
R-Bragg	99.993
Spacegroup	P-1
Scale	5.68882887e-006
Cell Mass	568.372
Cell Volume (Å^3)	834.63842
Wt% - Rietveld	100.000
Crystallite Size	
Cry size Lorentzian (nm)	3.6
Crystal Linear Absorption Coeff. (1/cm)	19.102
Crystal Density (g/cm^3)	1.131
PVII peak type	
FWHM = a + b/Cos(Th) + c Tan(Th)	
a	0.001135905
b	0.0008336294
c	0.0001
Exponent m = 0.6+ma+mb/Cos(Th)+mc/Tan(Th)	
ma	0.1015895
mb	0.1029966
mc	0.002438917
Lattice parameters	
a (Å)	8.0010524
b (Å)	11.8101211
c (Å)	8.8859681
alpha (°)	86.47793
beta (°)	94.669
gamma (°)	92.55243

Confined [BMIM][PF_6] in 2.3 nm pores at -84 °C

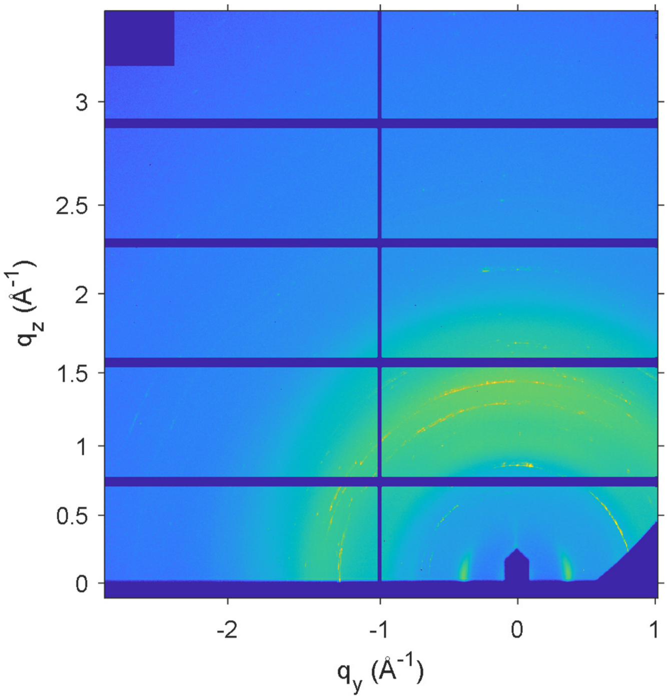

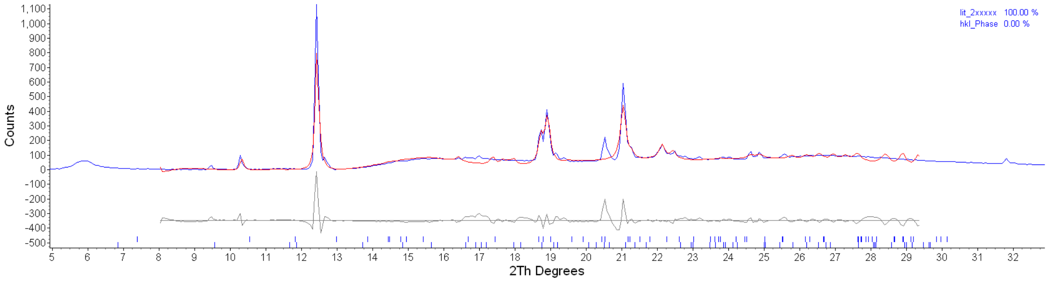

R-Values

Rexp : 10.05	Rwp : 27.05	Rp : 16.83	GOF : 2.69
Rexp` : 2.14	Rwp` : 5.75	Rp` : 7.02	DW : 0.76

Quantitative Analysis - Rietveld

Phase 1 : hkl_Phase	0.000 %
Phase 2 : lit_2xxxxx	100.000 %

Background

Chebychev polynomial, Coefficient	0	-108.6482
	1	62.89484
	2	-2.236589
	3	4.033432
	4	-38.1332
	5	1.047605

Instrument

Primary radius (mm)	217.5
Secondary radius (mm)	217.5

Corrections

Zero error	0.3497134
Specimen displacement	-0.7898925
LP Factor	0

Miscellaneous

Start X	8

Structure 2

Phase name	lit_2xxxxx
R-Bragg	99.997
Spacegroup	P-1
Scale	0.00031627151
Cell Mass	568.372
Cell Volume (Å^3)	723.43157
Wt% - Rietveld	100.000
Crystallite Size	
Cry size Lorentzian (nm)	7.8
Crystal Linear Absorption Coeff. (1/cm)	22.038
Crystal Density (g/cm^3)	1.305
PVII peak type	
FWHM = a + b/Cos(Th) + c Tan(Th)	
a	0.575721
b	0.5656161
c	0.9999999
Exponent m = 0.6+ma+mb/Cos(Th)+mc/Tan(Th)	
ma	0.0001
mb	0.0001
mc	0.06907023
Lattice parameters	
a (Å)	7.1088386
b (Å)	8.8532715
c (Å)	13.3699974
alpha (°)	91.67198
beta (°)	114.7952
gamma (°)	106.1454

Confined [BMIM][PF$_6$] in 8.2 nm pores at -130 °C

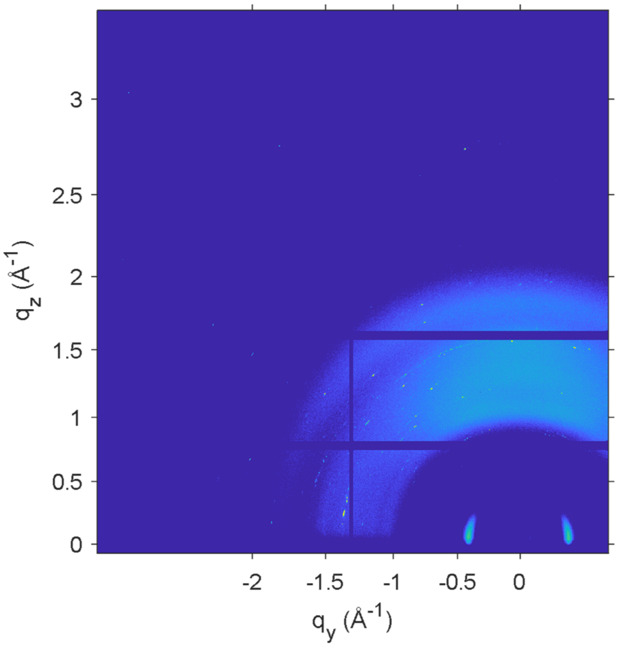

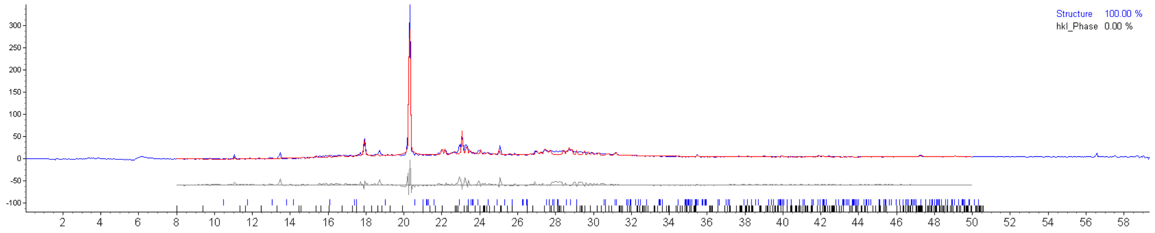

R-Values

```
Rexp : 26.10    Rwp : 25.31    Rp  : 17.65   GOF : 0.97
Rexp`: 53.89    Rwp`: 52.24    Rp` : 45.77   DW  : 1.26
```

Quantitative Analysis - Rietveld

```
   Phase 1  : hkl_Phase                       0.000 %
   Phase 2  : Structure                       100.000 %
```

Background

```
   Chebychev polynomial, Coefficient  0      4.300214
                                      1      1.907978
                                      2      -2.885172
                                      3      1.461686
                                      4      1.470159
                                      5      -1.30142
```

Instrument

```
   Primary radius (mm)                       217.5
   Secondary radius (mm)                     217.5
```

Corrections

```
   Zero error                                -0.6966527
```

LP Factor	0

Miscellaneous

Start X	8
Finish X	50

Structure 2

Phase name	Structure
R-Bragg	99.872
Spacegroup	P-1
Scale	5.54053237e-008
Cell Mass	568.372
Cell Volume (Å^3)	507.90148
Wt% - Rietveld	100.000
Crystallite Size	
Cry size Lorentzian (nm)	2959.2
Crystal Linear Absorption Coeff. (1/cm)	31.390
Crystal Density (g/cm^3)	1.858
Lattice parameters	
a (Å)	7.7730522
b (Å)	8.8879274
c (Å)	8.4929573
alpha (°)	96.83687
beta (°)	114.3848
gamma (°)	102.2719

Confined [BMIM][PF_6] in 8.2 nm pores at -61 °C

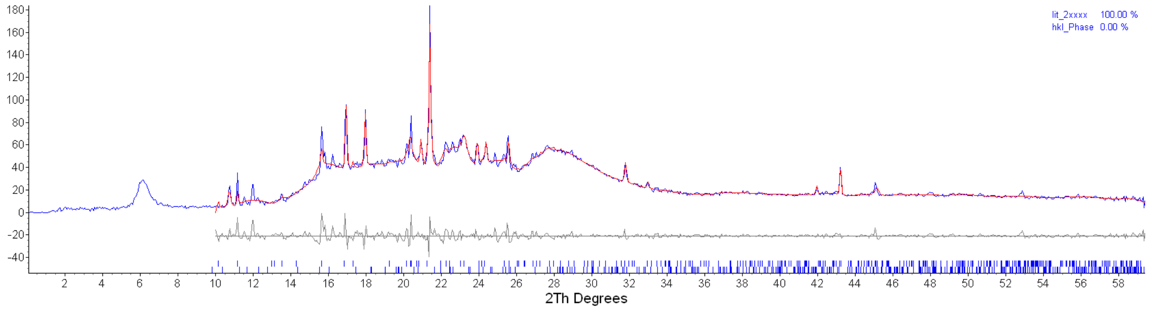

R-Values

Rexp : 11.30   Rwp : 8.89   Rp : 5.51   GOF : 0.79
Rexp`: 6.48    Rwp`: 5.10   Rp` : 4.55   DW : 1.46

Quantitative Analysis - Rietveld

Phase 1 : hkl_Phase	0.000 %
Phase 2 : lit_2xxxx	100.000 %

Background

One on X		98.00818
Chebychev polynomial, Coefficient	0	-12.84894
	1	30.66952
	2	-20.87548
	3	11.76158
	4	-4.900315
	5	-4.516647

Instrument

Primary radius (mm)	217.5
Secondary radius (mm)	217.5

Corrections

Zero error	0.3000172
Specimen displacement	0.5639539
LP Factor	0

Miscellaneous

Start X	10

Structure 2

Phase name	lit_2xxxx
R-Bragg	99.940
Spacegroup	P-1
Scale	5.16027343e-007
Cell Mass	568.372
Cell Volume (Å^3)	538.55715
Wt% - Rietveld	100.000

Crystallite Size
Cry size Lorentzian (nm) 166.7
Crystal Linear Absorption Coeff. (1/cm) 29.603
Crystal Density (g/cm^3) 1.752
PVII peak type
FWHM = a + b/Cos(Th) + c Tan(Th)
a 0.004504127
b 0.004481975
c 0.0004105741
Exponent m = 0.6+ma+mb/Cos(Th)+mc/Tan(Th)
ma 0.0001
mb 0.0001
mc 1.289897
Lattice parameters
a (Å) 7.5570020
b (Å) 9.2197380
c (Å) 9.0001039
alpha (°) 97.12184
beta (°) 114.8494
gamma (°) 102.6104

Site	Np	x	y	z	Atom	Occ	Beq
P1	2	0.18444	0.79488	0.44901	P	1	2.199
F1	2	0.35521	0.93965	0.57091	F	1	3.237
F2	2	0.29779	0.67516	0.50853	F	1	3.869
F3	2	0.12957	0.78229	0.59432	F	1	4.256
F4	2	0.07314	0.91488	0.38757	F	1	3.664
F5	2	0.24066	0.81036	0.30242	F	1	4.295
F6	2	0.01489	0.65083	0.32406	F	1	4.035
N1	2	0.75199	0.72952	0.65019	N	1	2.132
N2	2	0.78894	0.83706	0.89274	N	1	2.187
C1	2	0.69310	0.69370	0.46853	C	1	3.182
H1A	2	0.58390	0.72160	0.41060	H	1	4.816
H1B	2	0.67150	0.58060	0.42940	H	1	4.816
H1C	2	0.78460	0.75510	0.44440	H	1	4.816
C2	2	0.70855	0.83227	0.72967	C	1	2.227
H2	2	0.63220	0.89270	0.67830	H	1	2.685
C3	2	0.86449	0.66566	0.76617	C	1	2.503
H3	2	0.91630	0.58900	0.74360	H	1	3
C4	2	0.88720	0.73249	0.91775	C	1	2.55
H4	2	0.95780	0.71150	1.02240	H	1	3.079
C5	2	0.76590	0.92927	1.02293	C	1	2.779
H5A	2	0.71160	1.01120	0.97740	H	1	3.316
H5B	2	0.88280	0.98340	1.11980	H	1	3.316
C6	2	0.65000	0.82430	1.08181	C	1	2.424
H6A	2	0.70700	0.74480	1.13030	H	1	2.921
H6B	2	0.64200	0.89010	1.17210	H	1	2.921
C7	2	0.46470	0.73904	0.94394	C	1	2.645
H7A	2	0.47170	0.66970	0.85540	H	1	3.158
H7B	2	0.40830	0.81790	0.89270	H	1	3.158
C8	2	0.35100	0.63970	1.00890	C	1	3.3
H8A	2	0.40610	0.56120	1.05940	H	1	4.974
H8B	2	0.23360	0.58560	0.91560	H	1	4.974
H8C	2	0.34000	0.70840	1.09360	H	1	4.974

Confined [BMIM][PF_6] in 8.2 nm pores at 0.5 °C

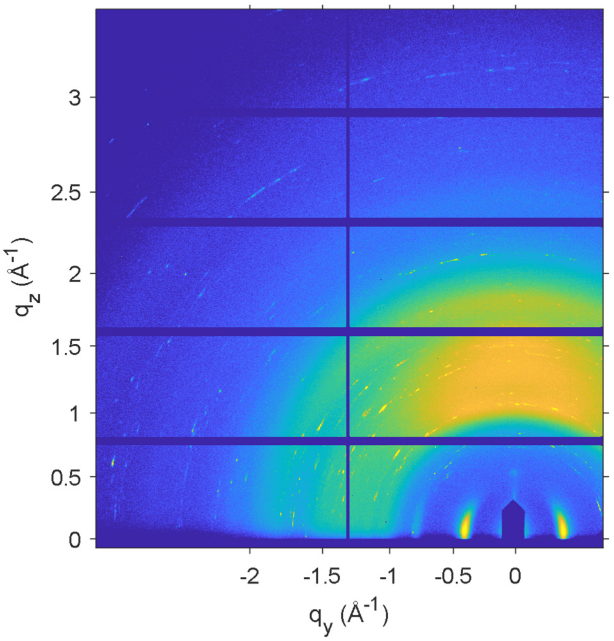

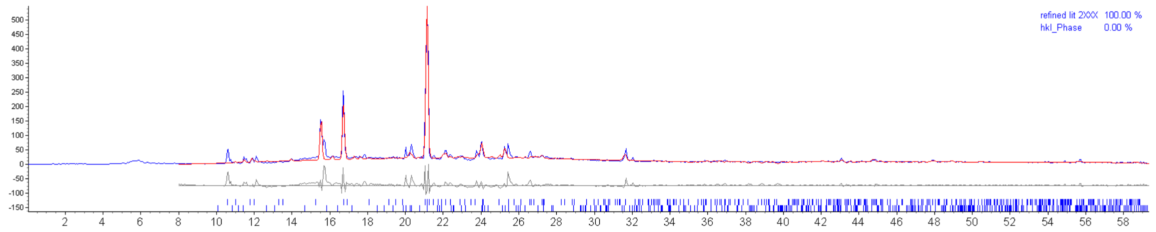

R-Values

Rexp : 17.77   Rwp : 25.05   Rp : 17.59   GOF : 1.41
Rexp`: 30.85   Rwp`: 43.49   Rp` : 37.88   DW : 1.05

Quantitative Analysis - Rietveld

Phase 1 : hkl_Phase   0.000 %
Phase 2 : "refined lit 2XXX"   100.000 %

Background

Chebychev polynomial, Coefficient	0	8.245037
	1	-3.276262
	2	-4.213524
	3	7.762794
	4	-1.826303
	5	-3.068859

Instrument

Primary radius (mm)   217.5
Secondary radius (mm)   217.5

Corrections

Zero error   0.8810527

LP Factor	0

Miscellaneous

Start X	8

Structure 2

Phase name	refined lit 2XXX
R-Bragg	17.724
Spacegroup	P-1
Scale	1.78411767e-006
Cell Mass	568.372
Cell Volume (Å^3)	592.27633
Wt% - Rietveld	100.000
Crystallite Size	
Cry size Lorentzian (nm)	10000.0
Crystal Linear Absorption Coeff. (1/cm)	26.918
Crystal Density (g/cm^3)	1.594
PVII peak type	
FWHM = a + b/Cos(Th) + c Tan(Th)	
a	0.0298965
b	0.01624297
c	0.02230486
Exponent m = 0.6+ma+mb/Cos(Th)+mc/Tan(Th)	
ma	20
mb	5
mc	5
Lattice parameters	
a (Å)	8.6283531
b (Å)	8.7861253
c (Å)	9.0291505
alpha (°)	95.67048
beta (°)	114.379
gamma (°)	103.2429

Confined [BMIM][PF_6] in 2.3 nm pores with [TMS-MIM][Cl] tethering at -49.5 °C

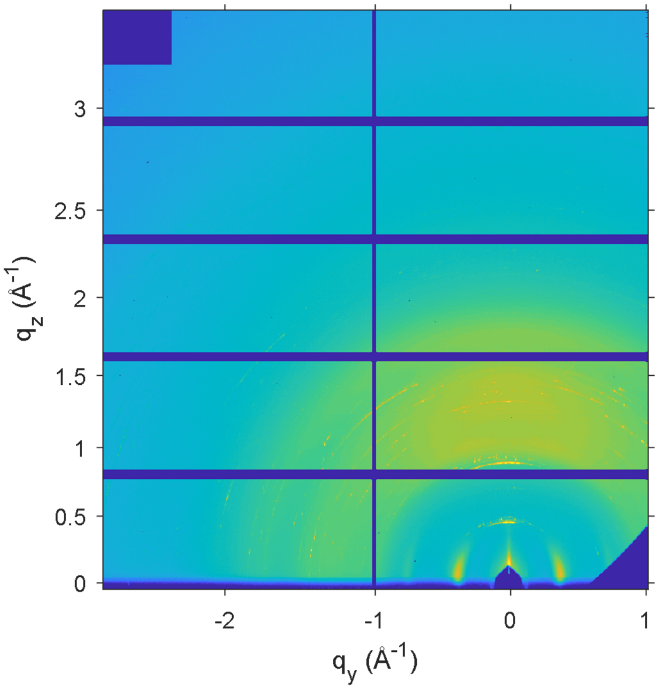

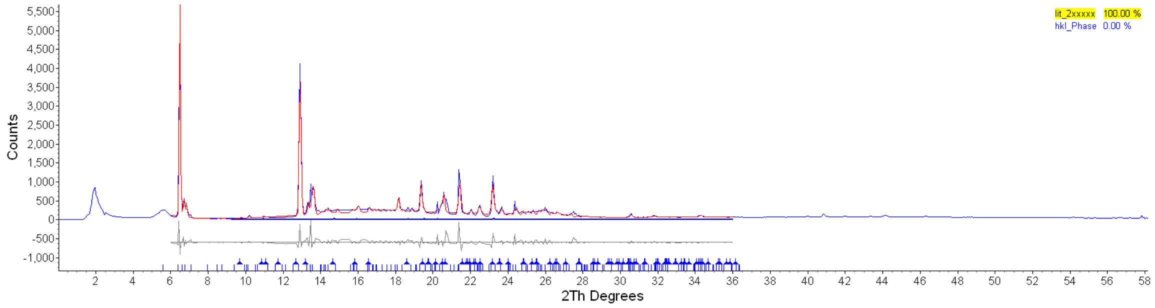

R-Values

```
Rexp : 5.36     Rwp : 19.90     Rp   : 14.44   GOF : 3.71
Rexp`: 9.29     Rwp`: 34.51     Rp`  : 29.61   DW  : 1.34
```

Quantitative Analysis - Rietveld

```
   Phase 1  : lit_2xxxxx                      100.000 %
   Phase 2  : hkl_Phase                       0.000 %
```

Background

```
   Chebychev polynomial, Coefficient  0       98.57399
                                      1       -11.32461
                                      2       -47.71616
                                      3       16.83724
                                      4       36.52009
                                      5       -38.94331
```

Instrument

```
   Primary radius (mm)                        217.5
```

Secondary radius (mm)	217.5

Corrections

Zero error	0.03551109
Specimen displacement	-0.1071728
LP Factor	0

Miscellaneous

Start X	6
Finish X	36

Structure 1

Phase name	lit_2xxxxx
R-Bragg	17.463
Spacegroup	P-1
Scale	2.12492909e-006
Cell Mass	568.372
Cell Volume (Å^3)	682.16180
Wt% - Rietveld	100.000
Crystallite Size	
Cry size Lorentzian (nm)	10000.0
Crystal Linear Absorption Coeff. (1/cm)	23.371
Crystal Density (g/cm^3)	1.384
PVII peak type	
FWHM = a + b/Cos(Th) + c Tan(Th)	
a	0.01222944
b	0.01039967
c	0.0001000001
Exponent m = 0.6+ma+mb/Cos(Th)+mc/Tan(Th)	
ma	20
mb	5
mc	5
Lattice parameters	
a (Å)	9.0242117
b (Å)	9.4764987
c (Å)	9.0506408
alpha (°)	94.63928
beta (°)	113.6794
gamma (°)	101.8333

Confined [BMIM]$[PF_6]$ in 2.3 nm pores with [TMS-MIM][Cl] tethering at -28.5 °C

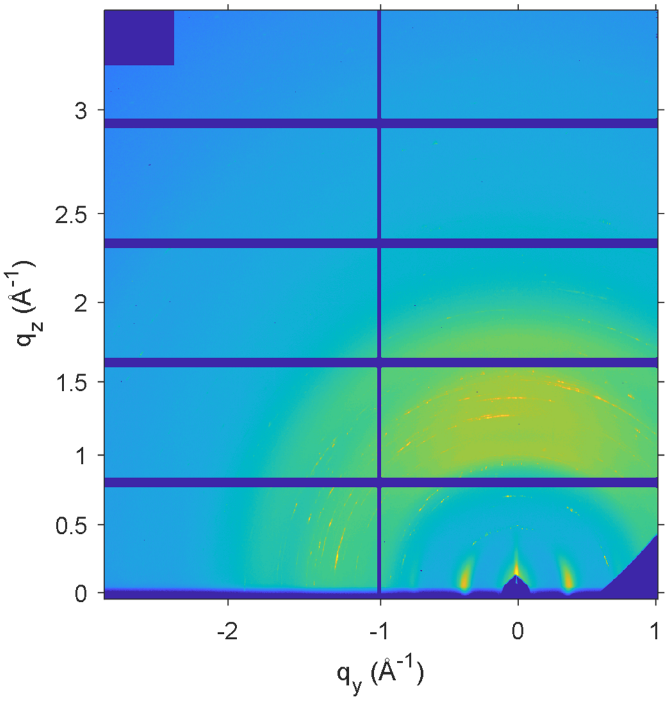

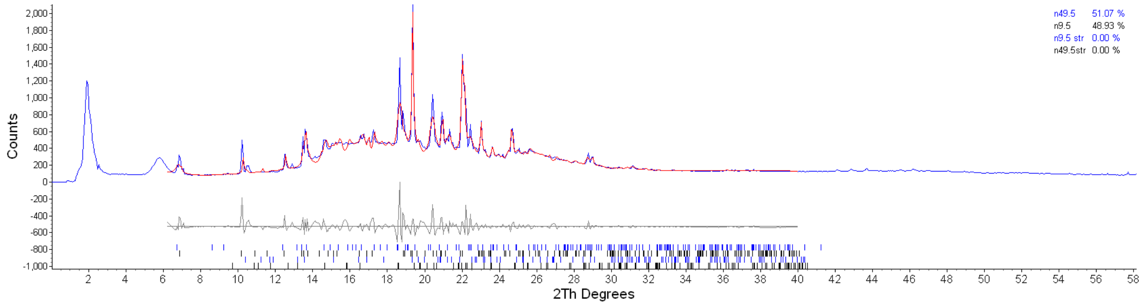

R-Values

```
Rexp : 3.43     Rwp : 11.69     Rp  : 7.67    GOF : 3.41
Rexp`: 6.64     Rwp`: 22.65     Rp` : 16.73   DW  : 1.43
```

Quantitative Analysis - Rietveld

Phase 1	: n49.5	51.069 %
Phase 2	: n9.5	48.931 %
Phase 3	: "n9.5 str"	0.000 %
Phase 4	: n49.5str	0.000 %

Background

Chebychev polynomial, Coefficient	0	140.9702
	1	14.24338
	2	-42.52262
	3	15.19745
	4	20.63974
	5	-19.61057

Instrument

Primary radius (mm)	217.5
Secondary radius (mm)	217.5

Corrections

Zero error	0.06335782
Specimen displacement	-0.07219221
LP Factor	0

Miscellaneous

Start X	6.2
Finish X	40

hkl Phase - 1 Pawley method

Phase name	n49.5
R-Bragg	1.458
Spacegroup	P-1
Cell Mass	568.372
Cell Volume (Å^3)	952.30732
Wt% - Rietveld	51.069
Crystallite Size	
Cry size Lorentzian (nm)	27.9
PVII peak type	
FWHM = a + b/Cos(Th) + c Tan(Th)	
a	0.01135947
b	0.01157184
c	0.009452699
Exponent m = 0.6+ma+mb/Cos(Th)+mc/Tan(Th)	
ma	20
mb	5
mc	5
Lattice parameters	
a (Å)	6.7339958
b (Å)	10.7386826
c (Å)	15.2708756
alpha (°)	106.548
beta (°)	115.4356
gamma (°)	87.31875

hkl Phase - 2 Pawley method

Phase name	n9.5
R-Bragg	1.355
Spacegroup	P-1
Cell Mass	568.372
Cell Volume (Å^3)	914.12259
Wt% - Rietveld	48.931
Crystallite Size	
Cry size Lorentzian (nm)	10000.0
PVII peak type	
FWHM = a + b/Cos(Th) + c Tan(Th)	
a	0.02343934
b	0.001744159
c	0.1141426
Exponent m = 0.6+ma+mb/Cos(Th)+mc/Tan(Th)	
ma	0.03117285
mb	0.02913543
mc	0.0001

Lattice parameters

a (Å)	9.1328053
b (Å)	7.9551910
c (Å)	13.3192964
alpha (°)	83.71658
beta (°)	78.62794
gamma (°)	102.5739

Confined [BMIM][PF_6] in 2.3 nm pores with [TSMIM][Cl] tethering at -9.5 °C

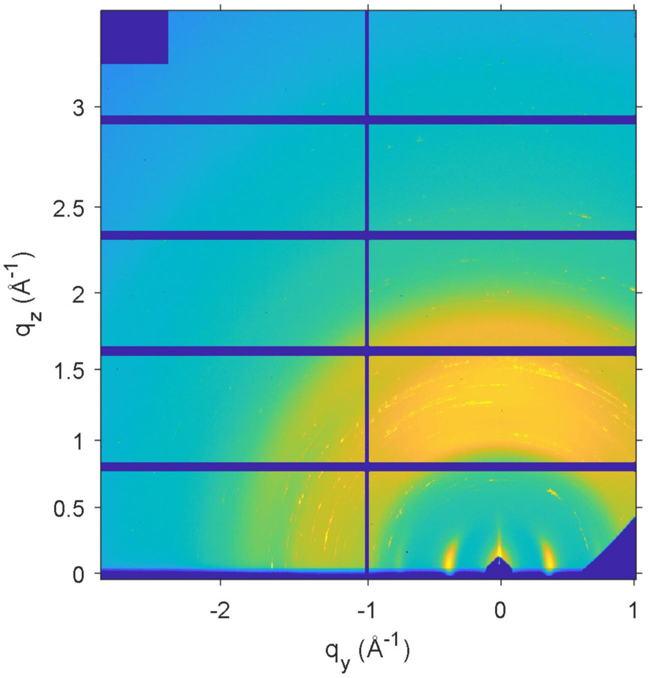

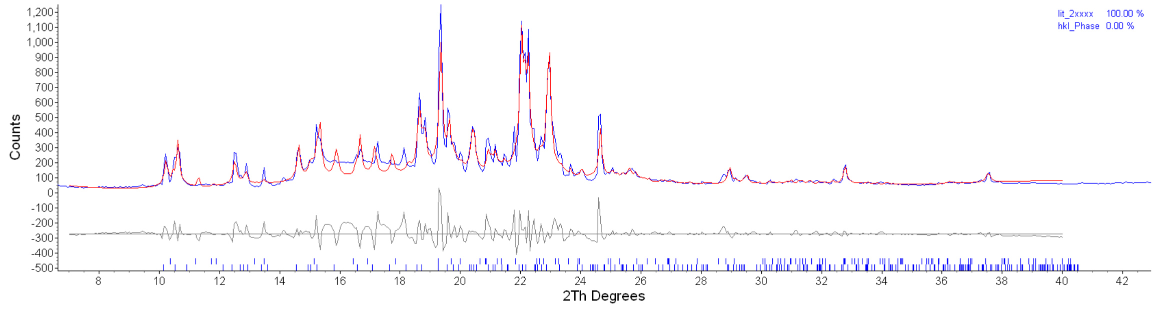

R-Values

Rexp : 6.14	Rwp : 19.71	Rp : 15.30	GOF : 3.21
Rexp`: 8.13	Rwp`: 26.12	Rp` : 21.27	DW : 1.15

Quantitative Analysis - Rietveld

Phase 1 : hkl_Phase	0.000 %
Phase 2 : lit_2xxxx	100.000 %

Background

Chebychev polynomial, Coefficient	0	44.70588
	1	22.50044
	2	6.337499
	3	7.557339
	4	10.46497
	5	-9.069654

Instrument

Primary radius (mm)	217.5
Secondary radius (mm)	217.5

Corrections

Zero error	0.0514826
Specimen displacement	-0.06454349
LP Factor	0

Miscellaneous

Start X	7
Finish X	40

Structure 2

Phase name	lit_2xxxx
R-Bragg	14.973
Spacegroup	P-1
Scale	5.63425085e-006
Cell Mass	568.372
Cell Volume (Å^3)	602.09058
Wt% - Rietveld	100.000
Crystallite Size	
Cry size Lorentzian (nm)	10000.0
Crystal Linear Absorption Coeff. (1/cm)	26.479
Crystal Density (g/cm^3)	1.568
PVII peak type	
FWHM = a + b/Cos(Th) + c Tan(Th)	
a	0.01894677
b	0.01380774
c	0.0001001927
Exponent m = 0.6+ma+mb/Cos(Th)+mc/Tan(Th)	
ma	19.99852
mb	4.99998
mc	0.0001173348
Lattice parameters	
a (Å)	8.7345916
b (Å)	8.9725120
c (Å)	8.9445080
alpha (°)	95.77493
beta (°)	115.3017
gamma (°)	102.9492

Confined [BMIM][PF_6] in 8.2 nm pores with [TSMIM][Cl] tethering at 25 °C

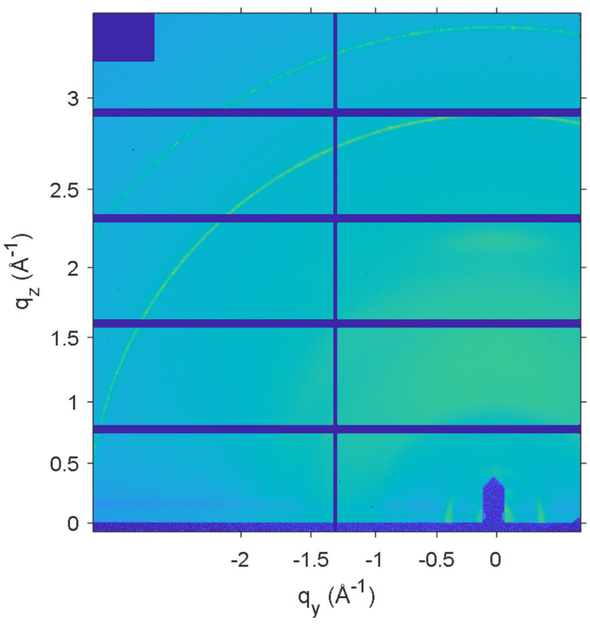

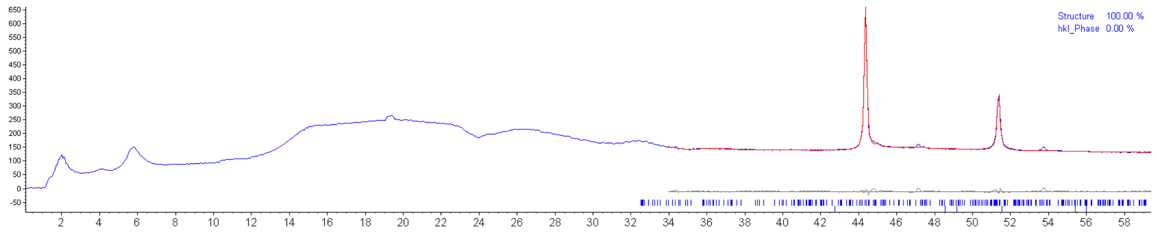

R-Values

```
Rexp : 7.79     Rwp : 1.42     Rp  : 0.94    GOF : 0.18
Rexp`: 43.18    Rwp`: 7.87     Rp` : 8.41    DW  : 0.82
```

Quantitative Analysis - Rietveld

```
   Phase 1  : hkl_Phase                        0.000 %
   Phase 2  : Structure                        100.000 %
```

Background

```
   Chebychev polynomial, Coefficient  0       132.8134
                                      1       -1.5377
                                      2       1.456767
                                      3       -3.316805
                                      4       3.711253
                                      5       -2.715081
```

Instrument

```
   Primary radius (mm)                        217.5
   Secondary radius (mm)                      217.5
```

Corrections

```
   Zero error                                 0.5587051
```

Specimen displacement	9.864444
LP Factor	0

Miscellaneous

Start X	34

Structure 2

Phase name	Structure
R-Bragg	0.178
Spacegroup	P-1
Scale	0.000146075982
Cell Mass	568.372
Cell Volume (Å^3)	606.12720
Wt% - Rietveld	100.000
Crystallite Size	
Cry size Lorentzian (nm)	10000.0
Crystal Linear Absorption Coeff. (1/cm)	26.303
Crystal Density (g/cm^3)	1.557
Preferred Orientation (Dir 1 : 0 1 0)	4.474878
(Dir 2 : 0 0 1)	0.8740383
Fraction of Dir 1	0.2149738
PVII peak type	
FWHM = a + b/Cos(Th) + c Tan(Th)	
a	0.6849915
b	0.0001
c	0.0001
Exponent m = 0.6+ma+mb/Cos(Th)+mc/Tan(Th)	
ma	0.5428876
mb	0.04136159
mc	0.1143809
Lattice parameters	
a (Å)	8.6076528
b (Å)	9.1331851
c (Å)	9.0189785
alpha (°)	94.59697
beta (°)	116.3858
gamma (°)	102.8464

Unconfined [BMIM][Cl] at -120 °C

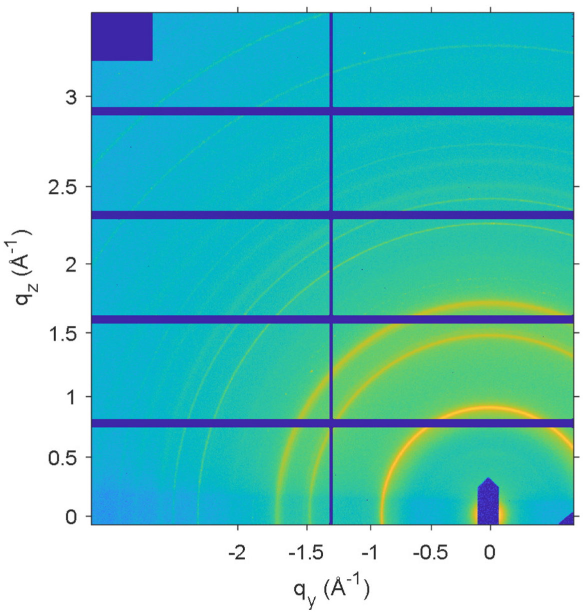

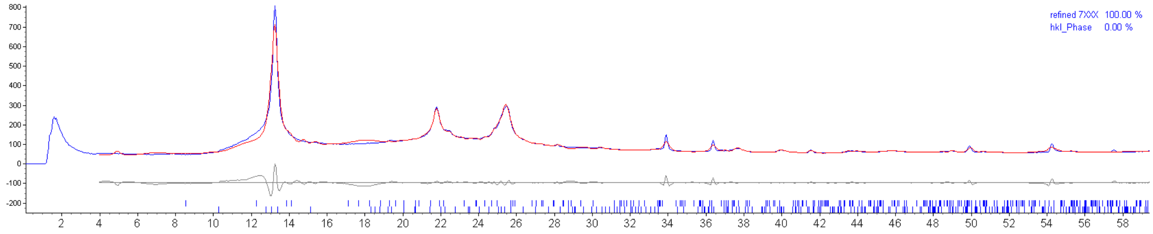

R-Values

Rexp : 8.65 Rwp : 6.93 Rp : 4.55 GOF : 0.80
Rexp`: 17.86 Rwp`: 14.31 Rp` : 10.43 DW : 0.25

Quantitative Analysis - Rietveld
Phase 1 : "refined 7XXX" 100.000 %
Phase 2 : hkl_Phase 0.000 %

Background
Chebychev polynomial, Coefficient 0 51.68264
1 0.4023822
2 0.1061073
3 17.41088
4 1.017197
5 -5.837033

Instrument
Primary radius (mm) 217.5
Secondary radius (mm) 217.5

Corrections
Zero error -0.05961568

Specimen displacement 10.07343
LP Factor 0

Miscellaneous

Start X 4

Structure 1

Phase name	refined 7XXX
R-Bragg	3.558
Spacegroup	P121/c1
Scale	3.80217761e-005
Cell Mass	698.695
Cell Volume (Å^3)	921.50462
Wt% - Rietveld	100.000
Crystallite Size	
Cry size Lorentzian (nm)	133.5
Crystal Linear Absorption Coeff. (1/cm)	31.848
Crystal Density (g/cm^3)	1.259
PVII peak type	
FWHM = a + b/Cos(Th) + c Tan(Th)	
a	0.3279577
b	0.3933629
c	1
Exponent m = 0.6+ma+mb/Cos(Th)+mc/Tan(Th)	
ma	0.0001
mb	0.0001
mc	4.999876
Lattice parameters	
a (Å)	11.4360533
b (Å)	10.0455773
c (Å)	8.8659273
beta (°)	115.2121

Site	Np	x	y	z	Atom	Occ	Beq
Cl1	4	0.25933	0.93049	0.09179	Cl	1	2.497
N1	4	1.07017	1.23886	0.76809	N	1	2.108
N3	4	1.31620	1.23743	0.91922	N	1	2.234
C2	4	1.18618	1.18274	0.88524	C	1	2.203
H2A	4	1.17680	1.11720	0.92900	H	1	2.369
C4	4	1.28205	1.33185	0.81932	C	1	2.4
H4A	4	1.35910	1.38210	0.82440	H	1	2.211
C5	4	1.12898	1.33249	0.72513	C	1	2.4
H5A	4	1.06240	1.37970	0.64300	H	1	2.606
C6	4	1.46890	1.20101	1.04160	C	1	2.985
H6A	4	1.45300	1.13160	1.09300	H	1	3.869
H6B	4	1.51600	1.26300	1.11500	H	1	3.79
H6C	4	1.53200	1.18170	0.99700	H	1	3.395
C7	4	0.90937	1.19944	0.68494	C	1	2.345
H7A	4	0.84950	1.26660	0.64200	H	1	2.685
H7B	4	0.88560	1.16560	0.75830	H	1	2.211
C8	4	0.88476	1.11324	0.55573	C	1	2.55
H8A	4	0.92310	1.14780	0.49000	H	1	2.921
H8B	4	0.94400	1.04370	0.60430	H	1	3
C9	4	0.71679	1.07988	0.45557	C	1	2.677
H9A	4	0.66020	1.14840	0.40420	H	1	2.685
H9B	4	0.67460	1.05250	0.52900	H	1	3

C10	4	0.69530	0.98570	0.33600	C	1	3.293
H10A	4	0.59100	0.96560	0.28000	H	1	4.974
H10B	4	0.75400	0.91500	0.39000	H	1	4.106
H10C	4	0.73700	1.01620	0.26300	H	1	3.869

Unconfined [BMIM][Cl] at 39 °C

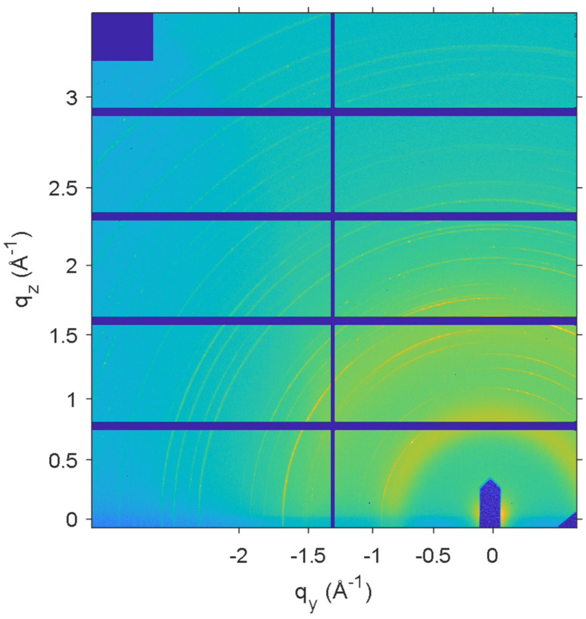

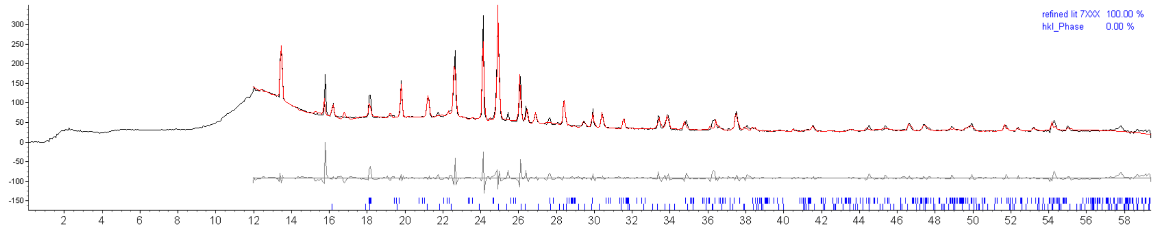

R-Values

Rexp : 11.28	Rwp : 10.02	Rp : 5.93	GOF : 0.89
Rexp`: 19.39	Rwp`: 17.23	Rp` : 11.76	DW : 1.51

Quantitative Analysis - Rietveld

Phase 1 : "refined lit 7XXX"	100.000 %
Phase 2 : hkl_Phase	0.000 %

Background

Chebychev polynomial, Coefficient	0	33.60476
	1	-21.46655
	2	22.57512
	3	-19.55366
	4	17.4492
	5	-13.76617

Instrument

Primary radius (mm)	217.5
Secondary radius (mm)	217.5

Corrections

Zero error	-0.1410659

Specimen displacement	10.26973
LP Factor	0

Miscellaneous

Start X	12

Structure 1

Phase name	refined lit 7XXX
R-Bragg	7.058
Spacegroup	P121/c1
Scale	1.17717394e-006
Cell Mass	698.695
Cell Volume (Å^3)	995.81316
Wt% - Rietveld	100.000
Crystallite Size	
Cry size Lorentzian (nm)	10000.0
Crystal Linear Absorption Coeff. (1/cm)	29.472
Crystal Density (g/cm^3)	1.165
PVII peak type	
FWHM = a + b/Cos(Th) + c Tan(Th)	
a	0.03372325
b	0.01503503
c	0.0001
Exponent m = 0.6+ma+mb/Cos(Th)+mc/Tan(Th)	
ma	20
mb	5
mc	5
Lattice parameters	
a (Å)	9.8734476
b (Å)	11.9506552
c (Å)	9.7346505
beta (°)	119.8932

Confined [BMIM][Cl] in 8.2 nm pores at -140 °C

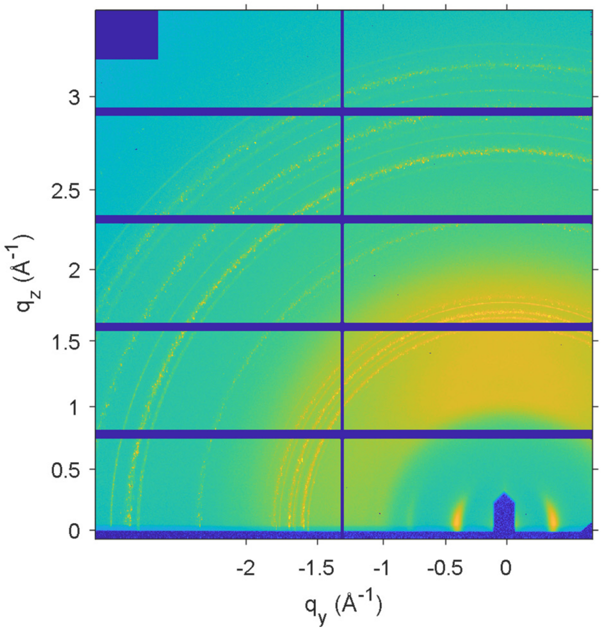

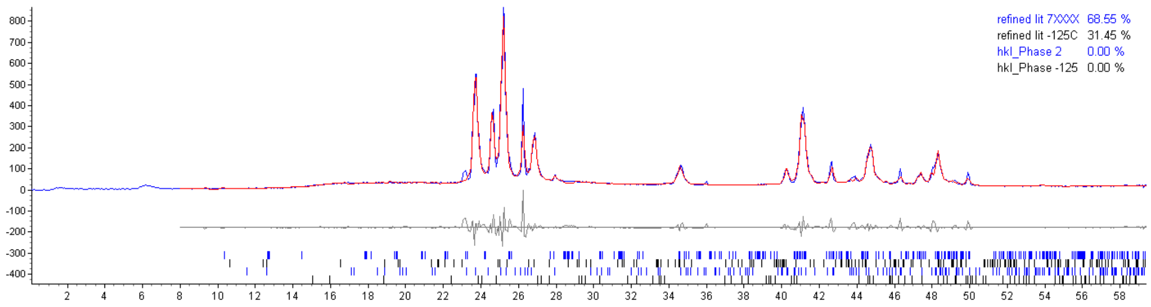

R-Values

```
Rexp : 11.51   Rwp : 13.07    Rp  : 9.28    GOF : 1.14
Rexp`: 16.02   Rwp`: 18.19    Rp` : 14.13   DW  : 1.29
```

Quantitative Analysis - Rietveld

```
   Phase 1  : "refined lit 7XXXX"        68.550 %
   Phase 2  : "hkl_Phase 2"              0.000 %
   Phase 3  : "refined lit -125C"        31.450 %
   Phase 4  : "hkl_Phase -125"           0.000 %
```

Background

```
   Chebychev polynomial, Coefficient  0      14.41823
                                      1      6.877887
                                      2      -7.418925
                                      3      4.886992
                                      4      3.021348
                                      5      -2.508542
```

Instrument

Primary radius (mm)	217.5
Secondary radius (mm)	217.5

Corrections

Zero error	-0.3487128
Specimen displacement	10.25972
LP Factor	0

Miscellaneous

Start X	8

Structure 1

Phase name	refined lit 7XXXX
R-Bragg	1.530
Spacegroup	P121/c1
Scale	9.48299318e-006
Cell Mass	698.695
Cell Volume (Å^3)	1017.08730
Wt% - Rietveld	68.550
Crystallite Size	
Cry size Lorentzian (nm)	21.0
Crystal Linear Absorption Coeff. (1/cm)	28.855
Crystal Density (g/cm^3)	1.141
PVII peak type	
FWHM = a + b/Cos(Th) + c Tan(Th)	
a	0.03486916
b	0.03423211
c	1
Exponent m = 0.6+ma+mb/Cos(Th)+mc/Tan(Th)	
ma	0.0001
mb	0.0001
mc	0.007677142
Lattice parameters	
a (Å)	9.8475827
b (Å)	12.2232233
c (Å)	9.7642267
beta (°)	120.0743

Structure 3

Phase name	refined lit -125C
R-Bragg	1.413
Spacegroup	C2
Scale	4.04575575e-006
Cell Mass	698.695
Cell Volume (Å^3)	1093.74127
Wt% - Rietveld	31.450
Crystallite Size	
Cry size Lorentzian (nm)	15.1
Crystal Linear Absorption Coeff. (1/cm)	26.833
Crystal Density (g/cm^3)	1.061
PVII peak type	
FWHM = a + b/Cos(Th) + c Tan(Th)	
a	0.02671795
b	0.0242638
c	0.5748069
Exponent m = 0.6+ma+mb/Cos(Th)+mc/Tan(Th)	
ma	0.0001

mb	0.0001
mc	0.003869578
Lattice parameters	
a (Å)	9.3938141
b (Å)	16.6190729
c (Å)	8.5140018
beta (°)	124.6267

Confined [BMIM][Cl] in 8.2 nm pores at -125 °C

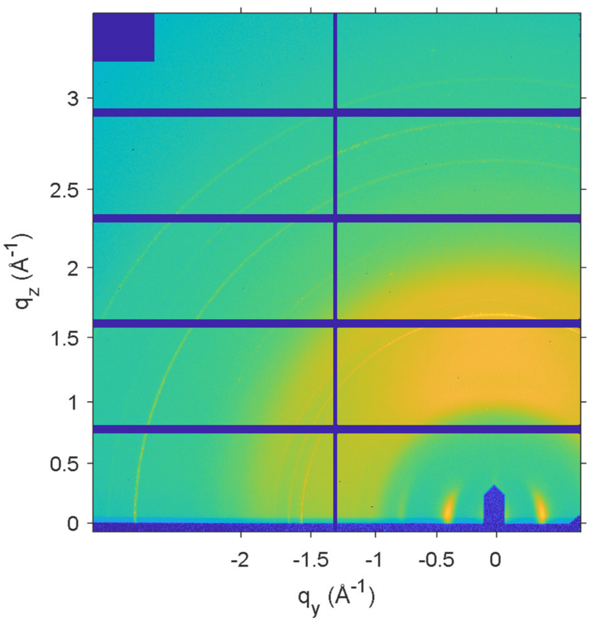

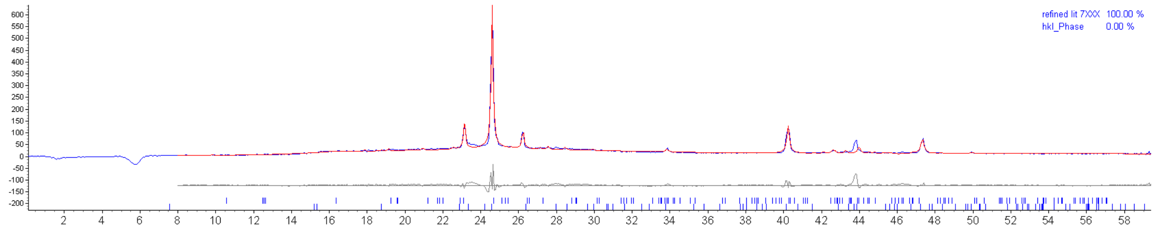

R-Values

```
Rexp : 19.56   Rwp : 15.19    Rp  : 11.13   GOF : 0.78
Rexp`: 34.95   Rwp`: 27.14    Rp` : 23.32   DW  : 0.80
```

Quantitative Analysis - Rietveld

```
   Phase 1  : "refined lit 7XXX"          100.000 %
   Phase 2  : hkl_Phase                   0.000 %
```

Background

```
   Chebychev polynomial, Coefficient  0       9.509502
                                      1       3.766029
                                      2       -7.25074
                                      3       5.770186
                                      4       -0.2623825
                                      5       -3.289541
```

Instrument

```
   Primary radius (mm)                        217.5
   Secondary radius (mm)                      217.5
```

Corrections

```
   Zero error                                 -0.2855491
```

Specimen displacement	9.911472
LP Factor	0

Miscellaneous

Start X	8

Structure 1

Phase name	refined lit 7XXX
R-Bragg	1.377
Spacegroup	C2
Scale	1.24560616e-005
Cell Mass	698.695
Cell Volume (Å^3)	1093.74136
Wt% - Rietveld	100.000
Crystallite Size	
Cry size Lorentzian (nm)	9999.0
Crystal Linear Absorption Coeff. (1/cm)	26.833
Crystal Density (g/cm^3)	1.061
PVII peak type	
FWHM = a + b/Cos(Th) + c Tan(Th)	
a	0.843499
b	0.5763494
c	0.0001
Exponent m = 0.6+ma+mb/Cos(Th)+mc/Tan(Th)	
ma	0.0001
mb	0.0001
mc	0.0001
Lattice parameters	
a (Å)	9.3938141
b (Å)	16.6190729
c (Å)	8.5140018
beta (°)	124.6267

References for Supporting Information

[1] A. Triolo, A. Mandanici, O. Russina, V. Rodriguez-Mora, M. Cutroni, C. Hardacre, M. Nieuwenhuyzen, H.-J. Bleif, L. Keller, M. A. Ramos, *The Journal of Physical Chemistry B* **2006**, 110, 21357.

[2] J. G. Huddleston, A. E. Visser, W. M. Reichert, H. D. Willauer, G. A. Broker, R. D. Rogers, *Green Chem.* **2001**, 3, 156.

[3] A. R. Choudhury, N. Winterton, A. Steiner, A. I. Cooper, K. A. Johnson, *Journal of the American Chemical Society* **2005**, 127, 16792.

[4] Y. Liu, Y. Zhang, G. Wu, J. Hu, *Journal of the American Chemical Society* **2006**, 128, 7456.

[5] T. Endo, T. Kato, K.-I. Tozaki, K. Nishikawa, *J. Phys. Chem. B* **2010**, 114, 407.

[6] B. Wu, R. Reddy, R. Rogers, *Solar Engineering* **2001**, 445.

[7] S. M. Dibrov, J. K. Kochi, *Acta Crystallographica Section C* **2006**, 62, o19.

[8] V. R. Koganti, S. E. Rankin, *The Journal of Physical Chemistry B* **2005**, 109, 3279.

[9] Y. He, D. Saang'onyo, F. Ladipo, B. L. Knutson, S. E. Rankin, *Industrial & Engineering Chemistry Research* **2019**, DOI: 10.1021/acs.iecr.9b03314.

[10] M. S. Rahman, J. Ambati, S. Joshi, S. E. Rankin, *Microporous Mesoporous Mater.* **2014**, 190, 74.

[11] a) S. Kumar, S. L. Jain, *New Journal of Chemistry* **2013**, 37; b) H. Valizadeh, M. Amiri, A. Shomali, F. Hosseinzadeh, *Journal of the Iranian Chemical Society* **2011**, 8, 495.

[12] W. L. Armarego, D. Perrin, D., *Purification of laboratory chemicals*, Butterworth-Heinemann, **1997**.
